# Regenerating the Injured Spinal Cord at the Chronic Phase by Engineered iPSCs‐Derived 3D Neuronal Networks

**DOI:** 10.1002/advs.202105694

**Published:** 2022-02-07

**Authors:** Lior Wertheim, Reuven Edri, Yona Goldshmit, Tomer Kagan, Nadav Noor, Angela Ruban, Assaf Shapira, Irit Gat‐Viks, Yaniv Assaf, Tal Dvir

**Affiliations:** ^1^ Shmunis School of Biomedicine and Cancer Research Faculty of Life Sciences Tel Aviv University Tel Aviv 6997801 Israel; ^2^ The Center for Nanoscience and Nanotechnology Tel Aviv University Tel Aviv 6997801 Israel; ^3^ The Department of Materials Science and Engineering Faculty of Engineering Tel Aviv University Tel Aviv 6997801 Israel; ^4^ Steyer School of Health Professions Sackler Faculty of Medicine Tel‐Aviv University Tel Aviv 6997801 Israel; ^5^ School of Neurobiology, Biochemistry and Biophysics Faculty of Life Sciences Tel Aviv University Tel Aviv 6997801 Israel; ^6^ Sagol School of Neuroscience Tel Aviv University Tel Aviv 6997801 Israel; ^7^ The Department of Biomedical Engineering Faculty of Engineering Tel Aviv University Tel Aviv 6997801 Israel; ^8^ Sagol Center for Regenerative Biotechnology Tel Aviv University Tel Aviv 6997801 Israel

**Keywords:** differentiation, induced pluripotent stem cells, spinal cord injury, tissue engineering, tissue implants, chronic phase injury, 3D neuronal network

## Abstract

Cell therapy using induced pluripotent stem cell‐derived neurons is considered a promising approach to regenerate the injured spinal cord (SC). However, the scar formed at the chronic phase is not a permissive microenvironment for cell or biomaterial engraftment or for tissue assembly. Engineering of a functional human neuronal network is now reported by mimicking the embryonic development of the SC in a 3D dynamic biomaterial‐based microenvironment. Throughout the in vitro cultivation stage, the system's components have a synergistic effect, providing appropriate cues for SC neurogenesis. While the initial biomaterial supported efficient cell differentiation in 3D, the cells remodeled it to provide an inductive microenvironment for the assembly of functional SC implants. The engineered tissues are characterized for morphology and function, and their therapeutic potential is investigated, revealing improved structural and functional outcomes after acute and chronic SC injuries. Such technology is envisioned to be translated to the clinic to rewire human injured SC.

## Graphical abstract

In a novel approach to regenerate the injured spinal cord (SC) at the chronic stage, induced pluripotent stem cells are encapsulated in a hydrogel to potentially form patient‐specific implants. The cells are differentiated to SC motor neurons and form 3D neuronal networks that bridge over the injured axons. The implants reduce inflammation, enhance nerve regeneration, and significantly improve locomotion.

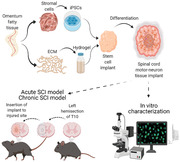

## 1 Introduction

Traumatic spinal cord injury (SCI) has an immediate and catastrophic impact on movement control and on all aspects of the patient's health and quality of life. The primary trauma injury causes direct damage, which often leads to death of cells, disruption to blood‐SC barrier, and degradation of the extracellular matrix (ECM). These processes initiate a secondary proinflammatory injury cascade, which leads to a progressive tissue damage resulting in the formation of a glial scar. Although the healthy neural tissue surrounding the injury site contains cues that may promote tissue repair,^[^ ^1^ ^]^ the lack of a permissive microenvironment for cell growth in the scar, along with the absence of ECM‐secreted axonal guidance molecules such as netrins and slits, results in very poor intrinsic regeneration potential and permanent neural dysfunction.^[^ ^2^, ^3^ ^]^

Furthermore, with time, the injured area expands, further challenging the ability for natural or intervened regeneration.^[^ ^4^ ^]^

Aiming to rewire the injured spinal cord (SC), researchers have suggested a variety of approaches including transplantation of different cell types or biomaterials into the injury site in the acute phase.^[^ ^5^, ^6^, ^7^, ^8^ ^]^ The implantation of Schwann cells,^[^ ^9^ ^]^ neural stem cells (NSCs) or neural progenitor cells (NPCs),^[^ ^10^, ^11^ ^]^ or mesenchymal stem cells^[^ ^12^ ^]^ has been investigated as a potential therapy for SC injury. However, two issues may jeopardize the success of the treatment, namely the immune response to allogenic or xenogeneic cells, which may promote cell rejection, and implantation of dissociated cells, not organized into a functional network.

To overcome the risk of rejection, induced pluripotent stem cells (iPSCs) may be used. In this approach, somatic cells from the patient are reprogrammed to become pluripotent and then differentiated to the desired cell lineage.^[^ ^13^ ^]^ The most common strategy in regeneration of the injured SC is not the direct application of the undifferentiated cells, but rather the transplantation of various iPSCs‐derived cell lines. Lu et al. inserted dissociated iPSC‐derived NSCs in a fibrin matrix two weeks after SCI induction. The cells were able to differentiate and interact with the host's neurons to form axons that extended through long distances of the white matter of the injured SC.^[^ ^14^ ^]^ In another work, iPSCs‐derived neurospheres were injected into the SC 9 days post SCI. Transplanted cells, which had differentiated in vivo into the three neural lineages without forming teratomas, participated in remyelination and improved locomotion.^[^ ^15^ ^]^ Although such a cell source may be relevant for regenerating the injured SC, injection of the cells into the injured area is not ideal. Once the dissociated cells in suspension or within biomaterial‐based carriers are injected into the injury site, energy is invested to form cell‐cell and cell‐matrix interactions for tissue formation and differentiation, and for integration with the healthy part of the SC.^[^ ^16^ ^]^ As the scar tissue, which is formed in the natural course of the pathophysiology, does not provide a supporting microenvironment for tissue assembly, massive cell death may occur. Therefore, insertion of a pre‐formed 3D neuronal network into the injury site and instead of the scar tissue may reduce the time required for regeneration and improve the efficacy of the treatment. However, the conditions for engineering functional 3D neural networks are still not fully known.^[^ ^17^ ^]^

We hypothesized that mimicking the embryonic development by applying a specific SC motor neuron differentiation protocol in a 3D dynamic microenvironment would provide the cells not only with differentiation cues, but also signals for appropriate tissue formation with natural hallmarks. We further hypothesized that assembling a functional neuronal network prior to implantation would increase the chances of functional engraftment.

**Figure 1:**
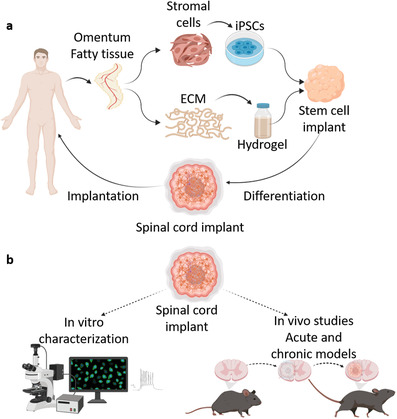
Schematics. a) The concept. Omental tissue is extracted from the patient. Then, cells and ECM are separated. While the cells are reprogrammed to become iPSCs, the ECM is processed into a thermoresponsive hydrogel. The iPSCs are then encapsulated within the omentum‐based hydrogel, to create stem cell implants. The implants are subjected to a 30‐day differentiation process, which mimics the embryonic SC development. The obtained SC neuron implants which are completely autologous, can then be implanted back into the patient. b) Study schematics. The differentiated SC motor neuron implants were first characterized in vitro. Next. the therapeutic potential of these implants was evaluated in hemisection acute and chronic SCI models. The molecular, behavioral, and anatomical aspects were investigated.

Recently, we developed iPSCs‐derived human tissue implants with the potential to perfectly match the immunological and cellular profile of a patient.^[^ ^18^ ^]^ In principle, in this approach, a small piece of fatty tissue biopsy is extracted from a patient and the cellular and acellular materials are separated. While the cells are reprogrammed to become iPSCs, the ECM is processed to become a personalized hydrogel.^[^ ^19^ ^]^ In this concept, following the encapsulation of patient‐specific iPSCs in the personalized hydrogel, efficient differentiation in a 3D permissive microenvironment is fostered, mimicking the embryonic development of the SC. The obtained functional SC implants may be introduced into the injury site to regain locomotion (**Figure** **1**a). Here, as a proof‐of‐concept, a porcine omentum‐based hydrogel was mixed with human iPSCs, the cells were specifically differentiated to SC motor neurons and SC implants were generated. The obtained implants were characterized for tissue morphology and function and their therapeutic potential was evaluated in both acute and chronic SC injury models in mice (Figure 1b).

## 2 Results and Discussion

### 2.1 Engineering Functional Spinal Cord Implants

**Figure 2:**
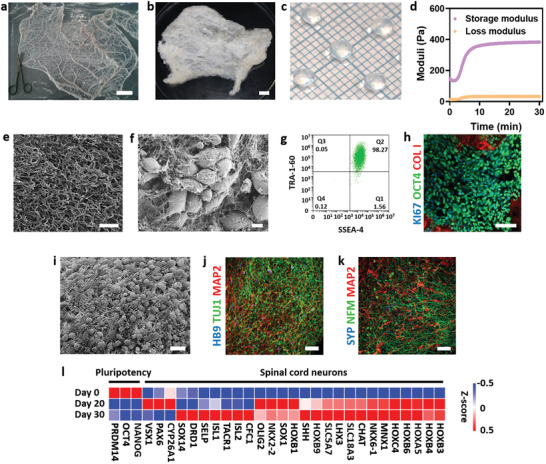
Generation and characterization of SC implants. a) Native omentum. b) Decellularized omentum. c) Omentum‐based hydrogel. d) Rheological properties of omentum‐based hydrogel. e) SEM imaging of acellular hydrogel. Scale bar = 2 µm. f) SEM imaging of iPSCs encapsulated within the hydrogel. Scale bar = 5 µm. g) Flow cytometry analysis of undifferentiated iPSCs (TRA‐1‐60, SSEA‐4) cultured for 3 days within the hydrogel. h) Immunofluorescence imaging of undifferentiated iPSCs cultured for 3 days within the omentum hydrogel and stained for Collagen (red), OCT4 (green), and KI67 (blue)I. Scale bar = 50 µm. i) SEM imaging of differentiated implants (day 30). Scale bar = 10 µm. j) Immunofluorescence of differentiated implants on day 30. Cells express a motoneuron specific marker (HB9; blue), a neuronal marker (TUJ1; green), and a dendritic marker (MAP2; red). Scale bar = 50 µm. k) Immunofluorescence of differentiated implants on day 30. Cells express markers for synapses (SYP; blue), neuronal intermediate filaments (NFM; green), and dendritic (MAP2; red). Scale bar = 50 µm. l) A heatmap of RNA‐seq expression Z‐scores computed for days 0, 20, and 30 of differentiation.

Porcine omental tissue was decellularized while preserving the extracellular matrix (ECM) (**Figure** **2**a,b and Figure S1, Supporting Information). The omentum is a fatty tissue enriched with blood vessels and sulfated glycosaminoglycans, and its ECM serves as a depot for stem cells in the body.^[^ ^20^ ^]^ These hallmarks endow this tissue with remarkable regenerative capabilities.^[^ ^21^ ^]^ The decellularized tissue was further processed into a thermoresponsive hydrogel (Figure 2c), which showed weak mechanical properties at room temperature, and physically crosslinked under physiological conditions (Figure 2c,d).

During natural embryonic development, at the blastocyst stage, the fibers of the ECM create niches, which support stem cell renewal, differentiation, and morphogenesis.^[^ ^22^, ^23^ ^]^ Scanning electron microscopy (SEM) images of the hydrogel revealed its fibrous structure, with an average fiber diameter of 91.7±33 nm (Figure 2e and Figure S2, Supporting Information). During embryonic development, pluripotent stem cells proliferate in a confined microenvironment prior to differentiation. To mimic this physiological process, human iPSCs colonies were mixed within an omentum hydrogel at a low concentration (Figure 2f). The cells expressed high pluripotency (TRA‐1‐60, SSEA4, OCT4) and proliferation (KI67) markers within the hydrogel (Figure 2g,h), and were allowed to proliferate and fill its volume. Then, to mimic the physiological process of neurogenesis, the iPSCs implants were subjected to a 30‐day SC motor neuron differentiation protocol within the 3D microenvironment. On day 30, the cells formed a high‐density 3D network within the entire implant (Figure 2i), expressing general and mid‐late neuronal markers, such as TUJ1 and MAP2, as well as the specific motor neuron marker HB9 (Figure 2j). Synapses and dendrites, as well as neurofilament formation within the implants, indicated the maturation and functionality of neuronal tissue (Figure 2k). Flow cytometry analyses indicated that more than 85% of the cells expressed the neuronal marker TUJ1, and more than 60% were also positive for HB9 (Figure S3, Supporting Information). Moreover, RNA sequencing at 3 different time points along the differentiation process (day 0, day 20, and day 30) revealed downregulation of pluripotency‐associated genes, and upregulation of neuronal and specifically SC motor neuron genes (Figure 2l). Multiple synergistic genes associated with the functions of neuronal activity and maturation were significantly enriched (Figure S4, Supporting Information).

**Figure 3:**
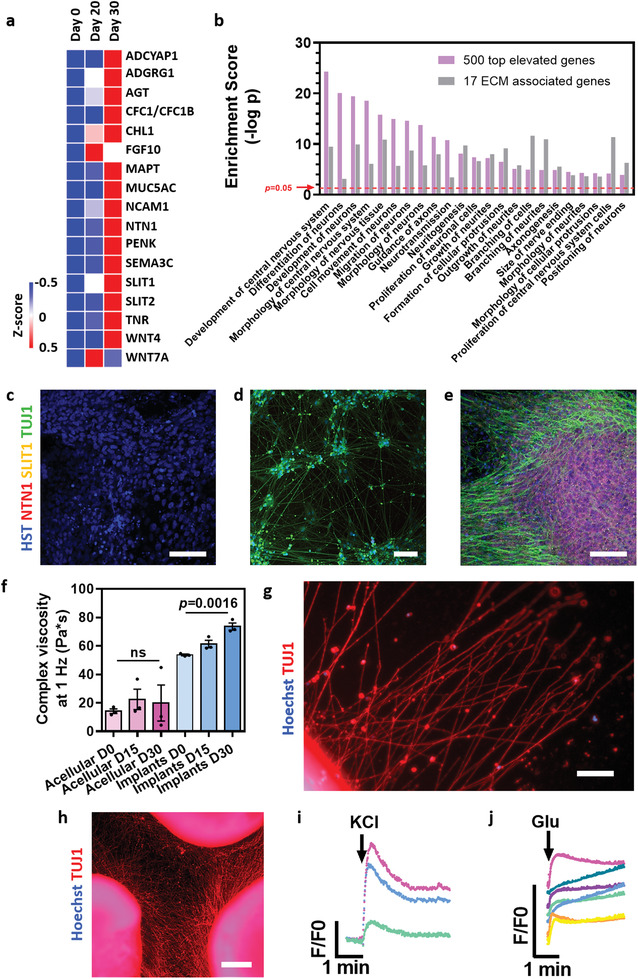
Engineered tissue ECM content and cell function. a) A heatmap of RNA‐seq expression Z‐scores computed for 17 secreted ECM proteins on days 0, 20, and 30 of differentiation. b) Bar chart showing the enriched functions on day 30 of differentiation (for the top elevated 500 genes), related to the 17 ECM genes in (a) as shown in Figure S5, Supporting Information. c–e) Expression of ECM‐associated proteins (NTN1 and SLIT1). TUJ1 is used to identify neurons. Scale bars = 50 µm. (c) iPSCs implants before differentiation. (d) iPSCs‐derived SC neurons, differentiated for 30 days on Matrigel. (e) iPSCs‐derived SC implants on day 30 of differentiation. f) Rheological properties of acellular hydrogel and implants on days 0, 15, and 30 of differentiation. g) Neurite outgrowth from day‐30 implant, cultured on a Matrigel‐coated surface for 72 h. Scale bar = 50 µm. h) Neurite‐branched network formed between 3 implants after 72 h. Scale bar = 500 µm. i) Calcium response of implants to depolarization by KCl. j) Calcium response of implants to stimulation by glutamate.

During embryonic development, the dynamic ECM plays a pivotal role in cell identity differentiation, maintaining tissue‐specific functions and tissue assembly. In addition to the initial matrix proteins, which were provided by the fabricated hydrogel throughout the SC differentiation and maturation, the cells secreted additional specific ECM components and soluble factors, which interacted with the existing matrix. This remodeling of the ECM composition during development and differentiation provides a new microenvironment, which is essential to support cell migration and promotes axonal growth/guidance and synaptogenesis. To investigate the new extracellular microenvironment generated during the differentiation process, we looked for the expression of neuronal ECM‐associated genes. Out of the 500 most elevated genes, the cells expressed 17 genes related to the production of essential ECM proteins that were not expressed by the undifferentiated cells (**Figure** **3**a). Further analysis revealed that functions that were enriched within the top increasing 500 genes are associated with neuronal tissue formation and function, including neuronal differentiation and development, guidance of axons, neuritogenesis and branching, neuronal migration, and neurotransmission. Such developmental and cellular processes must be regulated and guided by different ECM molecules.^[^ ^24^, ^25^ ^]^ Indeed, these functions were also unequivocally enriched within the 17 ECM genes (Figure 3b and Figure S5, Supporting Information). These functions are essential for the appropriate formation of the SC during embryonic development.^[^ ^24^ ^]^ Successful recapitulation of these cellular secretions and expression patterns suggests that the supplied microenvironment and the cells mutually affect each other to generate the appropriate 3D neuronal network. While on 2D surfaces most of the soluble proteins are secreted to the medium, in the hydrogel they are spatiotemporally accumulated within the 3D microenvironment, allowing them to interact with the initial ECM and to affect the encapsulated cells. To assess protein secretion and accumulation within the 3D microenvironment, cells differentiated on Matrigel, undifferentiated implants, and 30‐day implants were stained for the representative ECM proteins SLIT1 and NTN1, which play a key role in axon guidance and are extremely important for SC positioning.^[^ ^26^ ^]^ SLIT1 proteins prevent migration of the motor neurons towards the ventral floorplate thereby allowing them to stay in their correct columns.^[^ ^22^ ^]^ Netrins, on the other hand, are part of the larger laminin gene family and play an important role in guiding axons to the midline.^[^ ^27^, ^28^ ^]^ As shown, while the proteins were not detected within the day 0 implants nor on 2D Matrigel surfaces, they were highly expressed around cells within the hydrogel (Figure 3c–e). Synthesis and presentation of ECM proteins affect the function of the developing tissue. However, their accumulation within the 3D hydrogel also altered the microenvironment's mechanical properties. While the complex viscosity of acellular implants was not changed over time, the change in ECM protein type and amount significantly altered its biochemical content, increasing the complex viscosity (Figure 3f and Figure S6, Supporting Information).

Interaction between implants, or between implants and the healthy part of the tissue, is extremely important for efficient engraftment and for initiating regenerative processes in any neural tissue.^[^ ^29^ ^]^ To assess the ability of the implants to interact with their surrounding environment, implants were plated on a thin layer of Matrigel, and neurite outgrowth was observed (Figure 3g). Furthermore, when several implants were positioned at a distance of ≈1 mm from each other, a branched network was formed between them within 3 days (Figure 3h). After confirming the formation of the 3D neuronal network and the presence of dendrites and synapses, the implants’ neuronal electrical activity was monitored using calcium imaging. KCl is known to reliably depolarize neurons membranes leading to calcium ions influx into the cells. As shown, KCl stimulation revealed a significant increase in fluorescence, indicating the chemically‐induced effect (Figure 3i). Furthermore, the excitatory neurotransmitter glutamate known to activate SC motor neurons was able to induce a significant increase in calcium release (Figure 3j).

### 2.2 Treatment at the Acute Phase of the Injury

**Figure 4:**
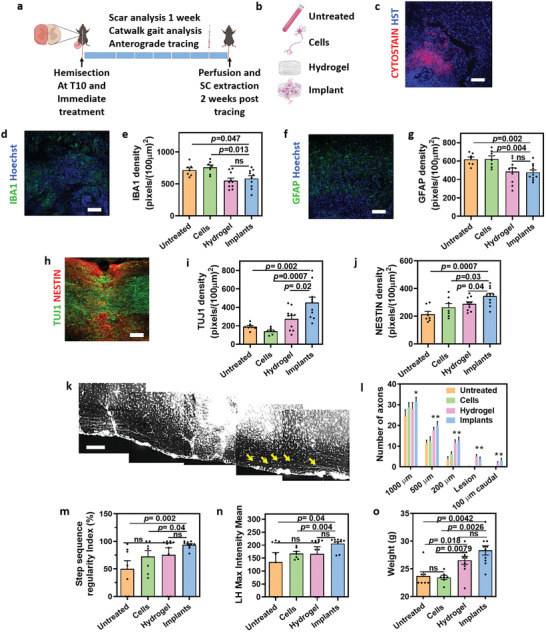
Acute SCI model. a) Schematics of the study. Mice were hemisected at T10, leaving the left hindlimb paralyzed. Treatment was administered immediately after the injury was induced. Mice were kept alive for 3 months, during which scar analyses, Catwalk gait analysis, and anterograde tracing were performed. Two weeks post tracing, the mice were transcardially perfused and the cords were extracted for analysis. b) Treatments administered: 3 control groups designated “Untreated”‐ animals treated with saline; “Cells”‐ animals treated with iPSCs‐derived SC neurons in suspension; “Hydrogel”‐ animals treated with acellular omentum‐based hydrogel; and an experimental group designated “Implants”‐ animals treated with iPSCs‐derived SC neuro implants. c–g) Cellular analysis of lesion site 7 days post‐treatment. (c) Engraftment of implants 7 days post‐implantation. Prior to implantation, the cells were labeled with CytoPainter (red). (d) Representative image of microglia (IBA1) expression in the “Implants” group. (e) Quantification of IBA1 density in the different groups. (f) Representative image of astrocytes (GFAP) in implants group. (g) Quantification of GFAP expression in the different groups. h–j) Cellular analysis of the lesion site 12 weeks post‐treatment. (h) Representative image of neural stem cells (NESTIN) and neurons (TUJ1) in the “Implants” group. (i) Quantification of TUJ1 density in the different groups. (j) Quantification of NESTIN density in the different groups. k) Montage of anterograde tracing of the “Implants” group. Yellow arrows indicate axons that were observed caudal to lesion site. l) Quantification of axons at different distances from the lesion, as labeled by anterograde tracing. m,n) Catwalk gait analysis parameters 12 weeks post‐treatment. (m) Degree of normal step sequence patterns‐ regularity index. (n) Maximum pressure (left hind max intensity mean) exerted by the left hind paw. o) Weight of mice 12 weeks post‐treatment. Untreated n = 7, cells n = 7, hydrogel n = 10, and implants n = 10. All scale bars = 100 µm.

After efficiently mimicking the embryonic SC development and engineering functional tissue implants, we sought to evaluate their therapeutic potential. Initially, as a proof of concept, an acute injury model in mice was chosen. Here, a complete left side hemisection at vertebrate T10 was performed with the right side of the SC remaining intact (**Figure** **4**a). Then, saline (untreated), 2D‐differentiated cell suspension in saline (cells), hydrogel without cells or the full implants (Figure 4b) were immediately inserted into the injury site and their ability to reduce inflammation and glial scar formation, promote neuroprotection and axonal regeneration and improve mice locomotion, were assessed. To compare between the engraftment of dissociated cells and that of the full implant, the cells were first pre‐labeled with a fluorescent dye. One week after treatment, the cells within the implant group were clearly detected at the lesion site, while cells that were applied in suspension were hardly observed, emphasizing the importance of a supporting microenvironment (Figure 4c and Figure S7, Supporting Information). After disruption of the blood‐SC barrier and hemorrhage, a secondary damage occurs and immune cells from the periphery or microglia from within the tissue migrate towards the injury site. This pro‐inflammatory environment results in additional neuronal death and massive accumulation of reactive astrocytes forming a glial scar. This process prevents further damage expansion; however, it inhibits spontaneous regeneration and propagates overall neuronal tissue degeneration.^[^ ^30^ ^]^ To assess the effect of the implants on the inflammatory process and progression within the injury site, the SC was extracted on day 7 post‐treatment, sectioned, and stained for microrglia (Iba1) and astrocytes (GFAP) markers. As shown, both the hydrogel and the implants significantly reduced the accumulation of both cell types (Figure 4c–g and Figure S8, Supporting Information) at the injury site. Moreover, the astrocytes detected within these groups were less reactive as observed by decreased GFAP protein expression, as well as lower expression of Ki67 within astrocytes (Figure S9, Supporting Information).

The lower level of inflammation observed on day 7 was translated to a more permissive environment with a significantly higher number of neurons (TUJ1) and neural progenitor cells (NESTIN) detected on week 12 in animals treated with the implants (Figure 4h–j and Figure S10, Supporting Information). The latter are cells with a capacity to differentiate to neurons or glial cells.^[^ ^31^ ^]^ Overall, within the implant group, the cells at the lesion site were organized in the direction of the SC tracts, bridging over the injury (Figure 4h). Such an organization of cells and essential ECM proteins may promote rewiring and regeneration across and within the injured SC.^[^ ^32^ ^]^

One important aspect of SC motor regeneration is the ability to transfer chemo‐electrical signals in axons crossing the injury site. To evaluate the potential of the implant to transfer a signal through the lesion site, mice were injected with an anterograde tracer molecule (TMRD) at the cervical level ipsilateral to the injury. Mice were kept for additional 2 weeks to allow the tracer to transport downstream through active neuronal axons. As shown, implants‐treated mice had a significantly higher number of axons that had reached and passed the lesion site, allowing new growth through the scar, whereas lower numbers of axons crossed the lesion in hydrogel‐treated mice, and none passed the lesion site in the other treatments (Figure 4k,l and Figure S11, Supporting Information).

The presence of functional axons along the SC tracts is essential for proper motor function. Therefore, we next sought to assess the ability of the implants to improve the locomotion of the treated mice by Catwalk gait analysis. As shown, all animals regained partial motor function, probably due to their ability to use the central pattern generator^[^ ^33^ ^]^ or due to spared contralateral motor fibers. However, the recovery was significantly improved in animals that were treated with the implants (Figure 4m,n and Movies S1–S4, Supporting Information). Compared to the untreated group, only animals treated with the implant showed a significantly better coordination, as judged by the higher regularity index (Figure 4m). Similarly, left hind maximal intensity, which indicates the ability of the animal to apply pressure to the injured foot, was significantly higher only in animals treated with the implant (Figure 4n). These improved behavioral parameters may be attributed to the synergistic effect of decreased inflammation and the presence of neurons in the lesion site, which are essential for regeneration. This recovery was also translated to a significantly higher gain in weight (Figure 4o), showing another aspect of the overall improved condition.

### 2.3 Treatment at the Chronic Phase of the Injury

Having demonstrated the ability of the implants to recover the injured SC in the acute phase, we moved on to assess their ability to regenerate the tissue in a more clinically relevant model. Immediately after the initial trauma, a secondary cascade of events, including bleeding and edema, takes place. These processes are temporary and their effect may be reversible.^[^ ^34^ ^]^ Therefore, treating patients at this stage, when the damage to the SC itself and its severity are still not clear, is irrelevant. Moreover, the process of engineering the implant may take several weeks, and it is highly unlikely that the injury will be treated immediately. Therefore, we sought to assess the potential of the implant to treat the injured SC once the damage has reached the chronic phase, where the scar is fully developed, and spontaneous behavioral recovery has reached its plateau.

**Figure 5:**
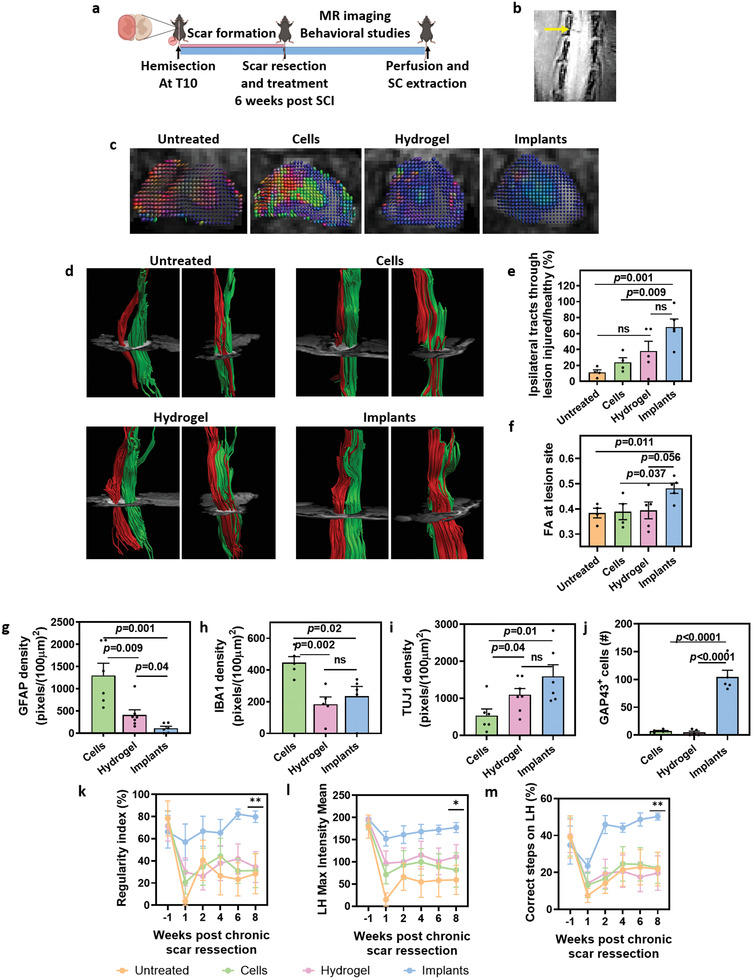
Chronic SCI model. a) Schematics of the study. Mice were hemisected at T10, leaving the left hindlimb paralyzed. Six weeks later, the lesion site was re‐exposed, scar was resected, and treatments were administered into the cavity. Mice were kept alive for additional 8 weeks post‐treatment, during which MRI and behavioral studies were performed. Eight weeks post‐treatment, the cords were extracted for histological analysis. b) Coronal T2W MRI of SC 5 weeks post initial SCI (1 week prior to scar resection). Yellow arrow indicates the complete hemisection performed on the left side of the SC. c–f) Diffusion tensor imaging at 4 weeks post‐treatment. (c) Glyph‐based visualization of diffusion tensor shown on the background of axial diffusion tensor images. Blue indicates fibers in the rostral‐caudal axis, red indicates right and left orientation both medial and lateral, while green indicates anterior to posterior orientation. (d) Fiber tractography reconstructed in the axial plane. Red fibers are ipsilateral to the initial hemisection, and green fibers are contralateral (unharmed). Left panel of each treatment: front view; right panel: side view. (e) Percentage of fibers passing through the lesion, normalized to the healthy side. (f) Fraction anisotropy (FA) measurements. g–j) Quantification of protein expression at the lesion site 8 weeks post‐treatment: (g) GFAP expression density. (h) IBA1 expression density. (i) TUJ1 expression density. (j) Number of GAP43 positive cells. k–m) Behavioral studies performed throughout the recovery period (post‐treatment). (k) Catwalk step sequence regularity index. (l) Maximum pressure exerted by left hindlimb. (m) Grid walk test: correct steps on the injured foot, out of all attempted steps. *p<0.05 was detected only between implants and untreated groups. **p<0.05 was detected between implants and all control groups. Untreated n = 4, cells n = 4, hydrogel n = 5, and implants n = 5.

For this purpose, we performed a complete hemisection as described for the acute phase. Six weeks after the initial SCI, the scar was ablated from the SC, and the same treatments were applied in the cavity (Figure 4b). Subsequently, the structural, biochemical, cellular, and behavioral parameters were assessed (**Figure** **5**a).

As shown, the complete hemisection could be easily detected by T2‐weighted MRI prior to scar resection (Figure 5b). To visualize the repair process of the SC tracts, we performed MRI utilizing diffusion tensor imaging (DTI) at 1‐ and 4‐weeks post‐treatment. The fractional anisotropy, extracted from DTI which provides valuable information on white matter integrity in general and of the SC in particular, is determined both by the axonal structural orientation and the state of myelin.^[^ ^35^ ^]^ The diffusion tensor in each voxel of an MRI image is represented by the principal eigenvector, indicating the fibers main orientation within the voxel.^[^ ^36^, ^37^ ^]^ As shown, one week after scar resection, the main diffusion orientation was random, rather than aligned along the SC axis (Figure S12, Supporting Information), which may indicate a severe injury.^[^ ^38^, ^39^ ^]^ However, at this stage of injury, it is likely that some of the detected damage was caused by edema after the scar resection, which could be completely or partially reversible.^[^ ^40^, ^41^ ^]^ Analysis at 4‐weeks after treatment revealed a major improvement in animals treated with the implants, as judged by the main diffusion orientation (indicated by their blue color) (Figure 5c). This improvement may be attributed to the integration of the implant and its ability to bridge between healthy axons above and below the lesion site. Streamline tractography was then used to visualize white matter fibers, their structural integrity, and the damage to the fiber bundle.^[^ ^36^, ^37^, ^42^ ^]^ Reconstructed neuronal tracts showed pathological changes in the SC at the lesion site for all animals (Figure 5d). However, tractography of untreated animals or animals treated with cells in suspension revealed higher displacement, deformation, and disruption in lesions. In these animals, only a few tissues or axons passing across the injured site were revealed, along with gradual Wallerian degeneration above the level of scar resection. In contrast, animals treated with the implants exhibited better preservation or restoration of the injured tracts, as judged by the number of tracts passing the lesion site (Figure 5d, Figure S13 and Movies S5–S8, Supporting Information). We next quantified the amount of ipsilateral nerve fibers crossing the injury site, and compared it to the fibers on the same levels on the healthy side. As shown, implants‐treated mice had significantly higher survival and/or regrowth of axons through the lesion, compared to untreated and cell‐treated animals (Figure 5e). We next analyzed the fractional anisotropy, which depends on the water diffusivity in the extracellular space along the axons.^[^ ^36^, ^37^ ^]^ Compared to all other treatments, animals treated with implants revealed significantly higher values at the lesion site (Figure 5f), indicating stronger anisotropy and a higher number of complete nerve fibers.

We next sought to analyze the cellular content at the injury site after a longer recovery period. The extensive damage caused by the removal of the scar tissue, with no further insertion of a supporting material, left a substantial cavity in several of the untreated animals. These SCs could not be extracted and processed in one piece, preventing quantification of the cellular content at the injury site of these animals and reliable analysis of this group. Comparison between the cells, hydrogel, and implant treatments revealed a significantly reduced chronic inflammation in animals treated with the implants, as evident by the lower density of reactive GFAP‐positive astrocytes (Figure 5g and Figure S14, Supporting Information). Microglia presence was also significantly reduced by both the hydrogel and implant treatments compared to the cells only group (Figure 5h and Figure S14, Supporting Information). As shown, significantly higher numbers of neurons were found both in hydrogel and implants‐treated mice (Figure 5i and Figure S14, Supporting Information). However, higher expression of GAP43, a marker associated with sprouting of axons in the development and regeneration of the SC, was only detected in the implant‐treated animals (Figure 5j and Figure S14, Supporting Information). This may suggest active axonal sprouting at the lesion site, which may gap the healthy axons on both sides of the lesion.

The potential of the implants to promote functional recovery was validated by sensorimotor function. During the experiment, mice were subjected to behavioral studies including Catwalk gate analysis and grid walk test. The coordination of mice, represented by step sequence regularity index, improved over time and reached its full potential 6 weeks after treatment (Figure 5k). Furthermore, higher pressure placed on the injured foot was detected already at one‐week post‐implantation in implant‐treated animals and was maintained throughout the experiment (Figure 5l). Finally, motoric and sensory recovery was observed in these animals, as indicated by less missed steps on the injured foot in grid walk analysis (Figure 5m). The significant recovery in the implant group, detected in the sensorimotor tests already on week 4 post‐treatment (Figure S15, Supporting Information) is in agreement with the results of the DTI obtained on that week, revealing intact fibers crossing the injury site (Figure 5d).

## 3 Summary and Conclusions

In summary, we report a new tissue engineering technology to treat the injured SC at the chronic stage. In this approach, the embryonic development of the SC was recapitulated by using an ECM‐based hydrogel, providing initial support material for iPSCs culture and expansion. Throughout the in vitro cultivation stage, the cells and the hydrogel showed a synergistic effect, mimicking the process of SC formation in the embryo. The processed hydrogel supported the efficient human iPSCs differentiation in 3D by providing the cells with an adequate microenvironment. Subsequently, during the differentiation, cells at different developmental stages continuously remodeled the hydrogel by secreting specific neuronal ECM proteins, providing an inductive microenvironment for cell‐cell and cell‐matrix interactions. Overall, this dynamic microenvironment, supplying different biochemical cues for the distinct developmental stages promoted functional SC implants assembly.

After assessing the morphology and function of the engineered tissues in vitro, their therapeutic effect was studied in vivo, first in the acute phase of SC injury. The implants enriched the hindered region with biochemical and mechanical cues to attract progenitor cells, supported cell survival and engraftment and reduced inflammation and gliosis at the lesion site, and overall improved the locomotion of the treated animal. The potential of the implants to treat the injured SC in the chronic phase, a more clinically relevant timeline, was then investigated. At this stage, the scar is fully developed and spontaneous behavioral recovery has reached its plateau. After scar tissue resection and insertion of the implants, the anatomy and tissue morphology were evaluated by MRI, indicating strong anisotropy and a high number of complete nerve fibers in the SC. Analysis of the cellular content within the lesion site indicated reduced inflammation levels and a higher number of neurons with elevated expression of markers associated with sprouting of axons during development and regeneration. These results were translated into a significantly higher level of behavioral functional recovery, as judged by the sensorimotor functional analyses.

Looking forward, after overcoming strict regulation challenges, the reported technology may be relevant for treating paralyzed human patients. In this concept, by taking a small biopsy from the patient, a personalized hydrogel can be produced and patient‐specific iPSCs can be generated to obtain personalized SC implants. The ability of the implants to replace the resected scar tissue and rewire the injured SC of humans may represent a novel personalized cell therapy approach.

## 4 Experimental Section

### Omentum Hydrogel Formation

Pig omenta (Kibutz Lahav, Israel) were washed with phosphate‐buffered saline (PBS) and major blood vessels were removed. The samples were then moved to a hypotonic buffer (10 mm Tris, 5 mm ethylenediaminetetraacetic acid (EDTA), and 1 µm phenylmethanesulfonyl‐fluoride (PMSF), pH 8.0) for 1 h. Next, tissues were frozen and thawed 3 times using the same buffer. The tissues were washed gradually with 70% ethanol and 100% ethanol for 30 min each. Lipids were extracted by three 30 min washes of 100% acetone, followed by 24h incubation in a 60/40 (v/v) hexane: acetone solution (3 changes). The defatted tissue was washed in 100% ethanol for 30 min and incubated overnight at 4 °C in 70% ethanol. Then, the tissue was washed four times with PBS (pH 7.4) and incubated in 0.25% Trypsin–EDTA (Biological Industries, Beit HaEmek, Israel) overnight. The tissue was washed thoroughly with PBS and incubated with 1.5 m NaCl for 24 h (3 changes), followed by washing in 50 mm Tris (pH 8.0), 1% triton‐X100 (Sigma) solution for 1 h. The decellularized tissue was washed in PBS followed by double distilled water and then frozen (−20 °C) and lyophilized.

After lyophilization, decellularized omentum was ground into powder (Wiley Mini–Mill, Thomas Scientific, Swedesboro, NJ)). Dry, milled omentum was enzymatically digested for 96 h at room temperature in a 1 mg mL^−1^ solution of pepsin (Sigma, 4000 U mg^−1^) in 0.1 m HCl, with stirring. Subsequently, the pH was adjusted to 7.4 using either DMEM/F12×10 or PBS X10 (Biological industries). The final concentration of decellularized omentum in the titrated solution was 1.5% (w/v). At least 10 pigs omenta were used.

### Culturing Undifferentiated iPSCs

iPSCs were generated from omental stromal cells and were a kind gift from Dr. Rivka Ofir, Ben Gurion University. The undifferentiated cells were cultivated on culture plates, pre‐coated with Matrigel (BD, New Jersey), diluted to 250 µg mL^−1^ in DMEM/F12 (Biological Industries), or cultured within the omentum hydrogel. All cells were cultured at 37 °C with 5% CO_2_. Undifferentiated iPSCs were maintained in NutriStem medium containing 0.1% Penicillin/Streptomycin (Biological Industries). Medium was replaced daily and cells were passaged weekly with 1 U mL^−1^ dispase (Stemcell Technologies, Vancouver, Canada) followed by mechanical trituration. iPSCs were seeded in small colonies in the presence of Y‐27632 (10 µm; Tocris, UK).

### Spinal Cord Motor Neurons Implant Generation and Differentiation

Dissociated iPSCs colonies were mixed with 1.5% omentum‐based hydrogel at equal volumes. Droplets of 3 µL were generated using a pipette. The implants were crosslinked at 37 °C on a damp towel for 30 min. Undifferentiated cells were cultured in Nutristem that was replaced daily, until 90% confluence was achieved. Cells were differentiated as previously described (2). Briefly, after achieving ≈90% confluence, medium was changed to Knockout/DMEM, supplemented with 15% Knockout Serum, 0.1% penicillin/streptomycin, 0.5% l‐glutamine, 1% non‐essential amino acids (Invitrogen), 10 µm *β*‐mercaptoethanol, 10 mm SB‐431542 (Tocris), 1 µm LDN‐193189 (Tocris), and 3 µm CHIR‐99021 and was gradually changed every 3 days to DMEM/F12 supplemented with N2 (Day 3 was ¾ Knockout/DMED and ¼ DMEM/F12 with N2 supplemented for the F12 portion only, day 6 was changed as ½ ½). On days 4 and 6, the motor neuron medium was supplemented with 1 µm retinoic acid and 1 µm purmorphamine (Tocris). On day 8, DMEM F/12 supplemented with N2, 30 ng mL^−1^ sonic hedgehog (R&D), and 1 µm retinoic acid was added to the cells (⅓ of the final volume, without changing medium). After day 10, the medium was changed to DMEM/F12 supplemented with N2, 0.1% P/S, 5 µg mL^−1^ BDNF (R&D), 200 µm ascorbic acid (Sigma), 1 µm purmorphamine (Tocris), and 1 µm retinoic acid. From day 15, 5 µm DAPT (Tocris) was also added, and purmorphamine concentration was decreased to 500 nm. Medium was changed every 3 days until day 30.

### Spinal Cord Hemisection

Hemisection was performed as previously described (5). Briefly, mice (20–30 g) were anesthetized with intraperitoneally‐injected ketamine (100 mg kg^−1^) and xylazine (16 mg kg^−1^) in PBS. The SC was exposed at the low thoracic to high lumbar area. After laminectomy, a complete left hemisection was made at T10 and the overlying muscle and skin were sutured. In acute phase injury, mice were immediately treated. The controls groups were: Untreated group which was treated with 10 uL saline; Cells group, treated with dissociated iPSCs‐derived SC neurons (day 30 of differentiation) suspended in 10 µL saline; and Hydrogel group that was treated with 0.75% pre‐crosslinked omentum‐based hydrogel. The tested treatment was applied to the Implants group which was treated with differentiated iPSCs‐derived SC neuron implants (day 30 of differentiation).

In chronic phase injury model, SCI was induced similarly to the acute phase. Six weeks after the initial SCI, animals were re‐anesthetized, and the lesion area was re‐opened. Scar was identified according to tissue color and texture and carefully resected. Treatments were applied in the cavity formed by the ablated scar. Mice were randomly assigned to the four groups and allowed to survive for 1 week to 3 months post‐injury.

All mice were treated according to the ethical regulations of Tel Aviv University. Permission was granted by the ethical committee, protocol number 04‐19‐047‐ “treatment of biological hydrogel and human neural cells on neuronal regeneration after acute and chronic SC injury in mouse model”.

### Catwalk Gait Analysis

Gait measurements were collected using the CatWalk XT system (Noldus Information Technology, The Netherlands). Data were transmitted to a computer and analyzed with the CatWalk XT software (version 10.6, Noldus). Each mouse was located on one side of the walkway and had to complete 3 compliant runs (variation < 60%; time < 5 s) to the other side. The coordination (regularity index) and the ability of the mouse to put pressure on the injured paw (left hind max intensity mean) were tested. The parameters were calculated for every run and the results were averaged for every time point per animal.

### Grid Walk

The mice were tested walking through a horizontal grid (1.2 × 1.2–cm grid spaces, 35 × 45–cm total area), one week prior to the treatment and on weeks 1, 2, 4, 6, and 8 post‐treatment. Each mouse was allowed to walk freely on the grid for 3 min. When the left hindlimb paw protruded entirely through the grid with all toes and heel, it was counted as a misstep. The number of missteps and total number of steps taken with the left hindlimb were both counted. The results were expressed as a percentage of correct steps on left hind paw (6).

### Magnetic Resonance Imaging (MRI)

MRI was performed on weeks 1 and 4 post chronic scar resection by a Bruker Biospec 7T/30 Scanner equipped with a 660mT/m gradient unit, using a cross coil configuration of 86mm transmissive volume coil and 10mm loop coil as a receiver. For untreated and cells groups N = 4, for hydrogel and implants groups N = 5. Animals were under 1–2% isoflurane in O_2_ anesthesia on a heating pad, with breathing monitored and body temperature maintained at 37 °C.

MRI protocol included the following methods: T2 weighted (T2w) images that were acquired using the rapid acquisition with relaxation enhancement (RARE) sequence and Diffusion Tensor Imaging (DTI) acquisition with a Diffusion‐Weighted Spin‐Echo Echo‐Planar‐Imaging pulse sequence (DW‐SE‐EPI). T2w acquisition was performed with the following parameters: TR = 8000 ms; effective TE: 30 ms, RARE factor 12 with 3 repetitions. 32 axial slices, 0.45 mm thick (no gaps), with in‐plane resolution of 0.15 mm^2^ covering the entire cerebrum, for 4 min. For DTI, performed for 5.5 min, the following parameters were used: TR/TE = 2500/19.2 ms, Δ/*δ* = 10/2.5 ms, 2 EPI segment, 30 gradient directions with b‐value at 1000 s mm^−2^ and three B0 images, 30 axial slices, 0.45 mm thick (no gaps), in‐plane resolution was 0.30 mm^2^. The total MRI protocol acquisition took ≈20 min. ExploreDTI software was used for DTI calculations and fiber tracking. The eigen‐components decomposed from the tensors were used for calculating fractional anisotropy maps. Regions of interest of the SC were manually segmented in each slice. Fiber tracking was employed for tract orientation with angle <30° and FA <0.15 and resolution of 2 2 2.

### Statistical Analysis

All statistical analyses were performed using GraphPad Prism 8.00 (GraphPad Software, Inc., USA). Data were shown as mean ± SEM (Standard Error of the Mean). Data were analyzed using Student's t‐test or one‐way analysis of variance (ANOVA) followed by Tukey's post‐hoc test. The values were considered significantly different at *p* < 0.05.

## Conflict of Interest

Tal Dvir is a co‐founder of Matricelf.

## Supporting information

Supporting Information

Supporting Movie 1

Supporting Movie 2

Supporting Movie 3

Supporting Movie 4

Supporting Movie 5

Supporting Movie 6

Supporting Movie 7

Supporting Movie 8

Supporting Movie 9

## Data Availability

The data that support the findings of this study are available in the supplementary material of this article.

## References

[1] Z. He , Y. Jin , Neuron 2016, 90, 437. 27151637 10.1016/j.neuron.2016.04.022

[2] F. T. Afshari , S. Kappagantula , J. W. Fawcett , Expert Rev. Mol. Med. 2009, 11, e37. 19968910 10.1017/S1462399409001288

[3] N. Zhang , H. Yan , X. Wen , Brain Res. Rev. 2005, 49, 48. 15960986 10.1016/j.brainresrev.2004.11.002

[4] A. E. Mautes , M. R. Weinzierl , F. Donovan , L. J. Noble , Phys. Ther. 2000, 80, 673. 10869130

[5] S. Usmani , A. Franceschi Biagioni , M. Medelin , D. Scaini , R. Casani , E. R. Aurand , D. Padro , A. Egimendia , P. Ramos Cabrer , M. Scarselli , M. De Crescenzi , M. Prato , L. Ballerini , Proc. Natl. Acad. Sci. U. S. A. 2020, 117, 25212. 32999065 10.1073/pnas.2005708117 PMC7568334

[6] Z.‐A. Yao , F.‐J. Chen , H.‐L. Cui , T. Lin , N. Guo , H.‐G. Wu , Neural Regener. Res. 2018, 13, 502. 10.4103/1673-5374.228756 PMC5900515 29623937

[7] F. Gelain , S. Panseri , S. Antonini , C. Cunha , M. Donega , J. Lowery , F. Taraballi , G. Cerri , M. Montagna , F. Baldissera , A. Vescovi , ACS Nano 2011, 5, 227. 21189038 10.1021/nn102461w

[8] Z. Álvarez , A. N. Kolberg‐Edelbrock , I. R. Sasselli , J. A. Ortega , R. Qiu , Z. Syrgiannis , P. A. Mirau , F. Chen , S. M. Chin , S. Weigand , E. Kiskinis , S. I. Stupp , Science 2021, 374, 848. 34762454 10.1126/science.abh3602 PMC8723833

[9] D. D. Pearse , F. C. Pereira , A. E. Marcillo , M. L. Bates , Y. A. Berrocal , M. T. Filbin , M. B. Bunge , Nat. Med. 2004, 10, 610. 15156204 10.1038/nm1056

[10] A. Marchini , A. Raspa , R. Pugliese , M. A. El Malek , V. Pastori , M. Lecchi , A. L. Vescovi , F. Gelain , Proc. Natl. Acad. Sci. U. S. A. 2019, 116, 7483. 30923117 10.1073/pnas.1818392116 PMC6462084

[11] E. S. Rosenzweig , J. H. Brock , P. Lu , H. Kumamaru , E. A. Salegio , K. Kadoya , J. L. Weber , J. J. Liang , R. Moseanko , S. Hawbecker , J. R. Huie , L. A. Havton , Y. S. Nout‐Lomas , A. R. Ferguson , M. S. Beattie , J. C. Bresnahan , M. H. Tuszynski , Nat. Med. 2018, 24, 484. 29480894 10.1038/nm.4502 PMC5922761

[12] A. E. Ropper , D. K. Thakor , I. Han , D. Yu , X. Zeng , J E. Anderson , Z. Aljuboori , S. ‐W. Kim , H. Wang , R. L. Sidman , R. D. Zafonte , Y. D. Teng , Proc. Natl. Acad. Sci. U. S. A. 2017, 114, E820. 28096400 10.1073/pnas.1616340114 PMC5293074

[13] K. Takahashi , K. Tanabe , M. Ohnuki , M. Narita , T. Ichisaka , K. Tomoda , S. Yamanaka , Cell 2007, 131, 861. 18035408 10.1016/j.cell.2007.11.019

[14] P. Lu , G. Woodruff , Y. Wang , L. Graham , M. Hunt , Di Wu , E. Boehle , R. Ahmad , G. Poplawski , J. Brock , L. S. B. Goldstein , M. H. Tuszynski , Neuron 2014, 83, 789. 25123310 10.1016/j.neuron.2014.07.014 PMC4144679

[15] O. Tsuji , K. Miura , Y. Okada , K. Fujiyoshi , M. Mukaino , N. Nagoshi , K. Kitamura , G. Kumagai , M. Nishino , S. Tomisato , H. Higashi , T. Nagai , H. Katoh , K. Kohda , Y. Matsuzaki , M. Yuzaki , E. Ikeda , Y. Toyama , M. Nakamura , S. Yamanaka , H. Okano , Proc. Natl. Acad. Sci. U. S. A. 2010, 107, 12704. 20615974 10.1073/pnas.0910106107 PMC2906548

[16] G. E. Salazar‐Noratto , G. Luo , C. Denoeud , M. Padrona , A. Moya , M. Bensidhoum , R. Bizios , E. Potier , D. Logeart‐Avramoglou , H. Petite , Stem Cells 2020, 38, 22. 31408238 10.1002/stem.3079

[17] M. Jorfi , C. D'Avanzo , D. Y. Kim , D. Irimia , Adv. Healthcare Mater. 2018, 7, 1700723. 10.1002/adhm.201700723 PMC5762251 28845922

[18] R. Edri , I. Gal , N. Noor , T. Harel , S. Fleischer , N. Adadi , O. Green , D. Shabat , L. Heller , A. Shapira , I. Gat‐Viks , D. Peer , T. Dvir , Adv. Mater. 2019, 31, 1803895. 10.1002/adma.201803895 30406960

[19] M. Shevach , R. Zax , A. Abrahamov , S. Fleischer , A. Shapira , T. Dvir , Biomed. Mater. 2015, 10, 034106. 25970726 10.1088/1748-6041/10/3/034106

[20] N. Soffer‐Tsur , M. Shevach , A. Shapira , D. Peer , T. Dvir , Biofabrication 2014, 6, 035023. 25162210 10.1088/1758-5082/6/3/035023

[21] V. Di Nicola , Regener. Ther. 2019, 11, 182. 10.1016/j.reth.2019.07.008 PMC6700267 31453273

[22] S. Wiese , A. Faissner , Exp. Neurol. 2015, 274, 90. 26028310 10.1016/j.expneurol.2015.05.018

[23] B. J. Dzamba , D. W. DeSimone , Curr. Top. Dev. Biol. 2018, 130, 245. 29853179 10.1016/bs.ctdb.2018.03.006

[24] A. Sagner , J. Briscoe , Development 2019, 146, dev182154. 31767567 10.1242/dev.182154

[25] D. A. C. Walma , K. M. Yamada , Development 2020, 147, dev175596. 32467294 10.1242/dev.175596 PMC7272360

[26] M. Kim , B. Bjorke , G. S. Mastick , Semin. Cell Dev. Biol. 2019, 85, 78. 29141180 10.1016/j.semcdb.2017.11.016 PMC5951725

[27] P. Mehlen , C. Delloye‐Bourgeois , A. Chédotal , Nat. Rev. Cancer 2011, 11, 188. 21326323 10.1038/nrc3005

[28] G. Lemke , Annu. Rev. Neurosci. 2001, 24, 87. 11283306 10.1146/annurev.neuro.24.1.87

[29] S. Kawabata , M. Takano , Y. Numasawa‐Kuroiwa , G. Itakura , Y. Kobayashi , Y. Nishiyama , K. Sugai , S. Nishimura , H. Iwai , M. Isoda , S. Shibata , J. Kohyama , A. Iwanami , Y. Toyama , M. Matsumoto , M. Nakamura , H. Okano , Stem Cell Rep. 2016, 6, 1. 10.1016/j.stemcr.2015.11.013 PMC4719132 26724902

[30] T. Yang , Y. J. Dai , G. Chen , S. S. Cui , Front. Cell. Neurosci. 2020, 14, 78. 32317938 10.3389/fncel.2020.00078 PMC7147295

[31] C. A. Messam , J. Hou , J. W. Berman , E. O. Major , Dev. Brain Res. 2002, 134, 87. 11947939 10.1016/s0165-3806(01)00325-x

[32] M. G. Tupone , M. D'angelo , V. Castelli , M. Catanesi , E. Benedetti , A. Cimini , Front. Bioeng. Biotechnol. 2021, 9. 10.3389/fbioe.2021.639765 PMC8012845 33816451

[33] L. Filli , M. E. Schwab , Neural Regener. Res. 2015, 10, 509. 10.4103/1673-5374.155425 PMC4424731 26170799

[34] A. V. Leonard , E. Thornton , R. Vink , J. Neurotrauma 2015, 32, 397. 25111333 10.1089/neu.2014.3543

[35] J. Cohen‐Adad , M ‐M. El Mendili , S. Lehéricy , P ‐F. Pradat , S. Blancho , S. Rossignol , H. Benali , Neuroimage 2011, 55, 1024. 21232610 10.1016/j.neuroimage.2010.11.089

[36] H. Johansen‐Berg , T. E. Behrens , Diffusion MRI: from quantitative measurement to in vivo neuroanatomy, Academic Press, Cambridge, Massachusetts 2013.

[37] P. Jones , K. Derek , Diffusion MRITheory, Methods, and Applications: Theory, Methods, and Applications, Oxford University Press, Oxford, England 2012.

[38] M. D. Budde , L. Janes , E. Gold , L. C. Turtzo , J. A. Frank , Brain 2011, 134, 2248. 21764818 10.1093/brain/awr161 PMC3155707

[39] D. Facon , A. Ozzana , P. Fillard , J.‐F. Lepeintre , C. Tornoux‐Facon , D. Ducreux , Am. J. Neuroradiol. 2005, 26, 1587. 15956535 PMC8149058

[40] J. H. Kim , D. N. Loy , Q. Wang , M. D. Budde , R. E. Schmidt , K. Trinkaus , S. ‐K. Song , J. Neurotrauma 2010, 27, 587. 20001686 10.1089/neu.2009.1063 PMC2867549

[41] E. Zakszewski , B. Schmit , S. Kurpad , M. D. Budde , J. Visualized Exp. 2015. 10.3791/52390 PMC4541495 25938297

[42] M. D. Budde , J. Annese , Front. Integr. Neurosci. 2013, 7, 3. 23378830 10.3389/fnint.2013.00003 PMC3561729

