# Supplementary material for: Regenerating the Injured Spinal Cord at the Chronic Phase by Engineered iPSCs‐Derived 3D Neuronal Networks
Source: Adv Sci (Weinh). 2022 Feb 7;9(11):2105694. doi: 10.1002/advs.202105694 (PMC9008789; doi:10.1002/advs.202105694)
Supplement: Supplementary file 1 — Supporting Information [file ADVS-9-2105694-s008.pdf]

ADVANCED SCIENCE
Open Access

Supporting Information

for *Adv. Sci.,* DOI: 10.1002/advs.202105694

Regenerating the injured spinal cord at the chronic phase by engineered iPSCs-derived 3D neuronal networks

*Lior Wertheim, Reuven Edri, Yona Goldshmit, Tomer Kagan, Nadav Noor, Angela Ruban, Assaf Shapira, Irit Gat-Viks, Yaniv Assaf, Tal Dvir**

Supporting Information

Regenerating the injured spinal cord at the chronic phase by engineered iPSCs-derived 3D neuronal networks

Lior Wertheim[1,2,3,#], Reuven Edri[1,#], Yona Goldshmit[1,4,#], Tomer Kagan[1], Nadav Noor[1], Angela Ruban[4], Assaf Shapira[1], Irit Gat-Viks[1], Yaniv Assaf[5,6], Tal Dvir[1,2,6,7,8]*

[1]Shmunis School of Biomedicine and Cancer Research, Faculty of Life Sciences, Tel Aviv University, Tel Aviv, Israel

[2]The Center for Nanoscience and Nanotechnology, Tel Aviv University, Tel Aviv, Israel

[3]The Department of Materials Science and Engineering, Faculty of Engineering, Tel Aviv University, Tel Aviv, Israel

[4]Steyer School of Health Professions, Sackler Faculty of Medicine, Tel-Aviv University, Tel Aviv, Israel

[5]School of Neurobiology, Biochemistry and Biophysics, Faculty of Life Sciences, Tel Aviv University, Tel Aviv, Israel

[6]Sagol School of Neuroscience, Tel Aviv University, Tel Aviv, Israel

[7]The Department of Biomedical Engineering, Faculty of Engineering, Tel Aviv University, Tel Aviv, Israel

[8]Sagol Center for Regenerative Biotechnology, Tel Aviv University, Tel Aviv, Israel

*Corresponding author: Tal Dvir (tdvir@tauex.tau.ac.il)

#Equal contribution

Materials and Methods

Omentum hydrogel formation

Omentum decellularization(*1*): Pig omenta (Kibutz Lahav, Israel) were washed with phosphate buffered saline (PBS) and major blood vessels were removed. The samples were then moved to a hypotonic buffer (10 mM Tris, 5 mM ethylenediaminetetraacetic acid (EDTA) and 1 μM phenylmethanesulfonyl-fluoride (PMSF), pH 8.0) for 1 hour. Next, tissues were frozen and thawed 3 times using the same buffer. The tissues were washed gradually with 70% ethanol and 100% ethanol for 30 min each. Lipids were extracted by three 30 min washes of 100% acetone, followed by 24h incubation in a 60/40 (v/v) hexane: acetone solution (3 changes). The defatted tissue was washed in 100% ethanol for 30 min and incubated overnight at 4 °C in 70% ethanol. Then, the tissue was washed four times with PBS (pH 7.4) and incubated in 0.25% Trypsin–EDTA (Biological Industries) overnight. The tissue was washed thoroughly with PBS and incubated with 1.5 M NaCl for 24 h (3 changes), followed by washing in 50 mM Tris (pH 8.0), 1% triton-X100 (Sigma) solution for 1 h. The decellularized tissue was washed in PBS followed by double distilled water and then frozen (-20 °C) and lyophilized.

Solubilization of omentum hydrogel: After lyophilization, decellularized omentum was ground into powder (Wiley Mini–Mill, Thomas Scientific, Swedesboro, NJ)). Dry, milled omentum was enzymatically digested for 96 h at room temperature in a 1 mg ml^{-1} solution of pepsin (Sigma, 4000 U mg^{-1}) in 0.1 M HCl, with stirring. Subsequently, the pH was adjusted to 7.4 using either DMEM/F12 X10 or PBS X10 (Biological industries). The final concentration of decellularized omentum in the titrated solution was 1.5% (w/v). At least 10 pigs omenta were used.

Culturing undifferentiated iPSCs

iPSCs were generated from omental stromal cells and were a kind gift from Dr. Rivka Ofir, Ben Gurion University. The undifferentiated cells were cultivated on culture plates, pre-coated with Matrigel™ (BD, New Jersey), diluted to 250 µg/mL in DMEM/F12 (Biological Industries, Beit HaEmek, Israel), or cultured within the omentum hydrogel. All cells were cultured at 37 °C with 5% CO_2. Undifferentiated iPSCs were maintained in NutriStem® (Biological Industries) medium containing 0.1% Penicillin/Streptomycin (Biological Industries). Medium was replaced daily and cells were passaged weekly with 1 U/mL dispase (Stemcell Technologies, Vancouver, Canada) followed by mechanical trituration. iPSCs were seeded in small colonies in the presence of Y-27632 (10µM; Tocris, UK).

Spinal cord motor neurons implants generation and differentiation

Dissociated iPSCs colonies were mixed with 1.5% omentum-based hydrogel at equal volumes. Droplets of 3 µL were generated using a pipette. The implants were crosslinked at 37 °C on a damp towel for 30 min. Undifferentiated cells were cultured in Nutristem that was replaced daily, until 90% confluence was achieved. Cells were differentiated as previously described (*2*). Briefly, after achieving ~90% confluence, medium was changed to Knockout/DMEM, supplemented with 15% Knockout Serum, 0.1% penicillin/streptomycin, 0.5% l-glutamine, 1% non-essential amino acids (Invitrogen), 10 µM β-mercaptoethanol, 10 mM SB-431542 (Tocris), 1 µM LDN-193189 (Tocris), and 3 µM CHIR-99021 and was gradually changed every 3 days to DMEM/F12 supplemented with N2 (Day 3 was ¾ Knockout/DMED and ¼ DMEM/F12 with N2 supplemented for the F12 portion only, day 6 was changed as ½ ½). On days 4 and 6, the motor neuron medium was supplemented with 1 µM retinoic acid and 1 µM purmorphamine (Tocris).

On day 8, DMEM F/12 supplemented with N2, 30 ng/mL sonic hedgehog (R&D) and 1 µM retinoic acid was added to the cells (⅓ of the final volume, without changing medium). After day 10, the medium was changed to DMEM/F12 supplemented with N2, 0.1% P/S, 5 µg/mL BDNF (R&D), 200 µM ascorbic acid (Sigma), 1 µM purmorphamine (Tocris) and 1 µM retinoic acid. From day 15, 5 µM DAPT (Tocris) was also added, and purmorphamine concentration was decreased to 500 nM. Medium was changed every 3 days until day 30.

Immunostaining and confocal imaging

Cellular implants were fixed in 4% formaldehyde, permeabilized with 0.05% (v/v) triton X-100 and blocked with PBS, 1% bovine serum albumin (BSA), 10% fetal bovine serum (FBS) and stained with the indicated primary antibodies followed by secondary antibodies (as presented in the Antibody list, Supporting Information). Cells and implants were imaged using an upright confocal microscope (Nikon ECLIPSE NI-E) and inverted fluorescence microscope (Nikon ECLIPSE TI-E). Images were processed and analyzed using NIS elements software (Nikon Instruments).

Neurite outgrowth assay

For neurite outgrowth assay, implants at day 30 of differentiation were placed on 250 µg/mL Matrigel™-coated plates. The constructs were cultured for 3 days before fixation in 4% formaldehyde and imaging using inverted fluorescence microscope (Nikon ECLIPSE TI-E).

RNA seq and bioinformatics analysis

RNA samples of implants on days 0, 20 and 30 were extracted using miRNeasy kit (Qiagen, Hilden, Germany) and were treated with DNase (Qiagen). Pooled samples from at least 2 different experimental replicates were quantified. Library quality control was performed using FASTQC (version 0.11.5) followed by quality and adapter trimming using Cutadapt, Version 1.1

(*3*). All reads were aligned to the *Homo sapiens* reference genome using the TopHat aligner (Trapnell) with a maximal mismatch parameter of 3 and minimum and maximum intron sizes of 70 and 500000, respectively. The raw expression levels were calculated using HTseq-count, version 0.6.1 (*4*). Reads per kilobase million (RPKM) values were then calculated based on the raw expression levels using annotations from Ensembel gene reference file (version GRCh38.87).

Functional enrichments were analyzed using the Ingenuity® Pathway Analysis (IPA®, Qiagen Bioinformatics, Redwood City, CA) software for the 500 protein-coding genes with the highest increasing expression based on the RPKM fold-change (day 30 of differentiation normalized to undifferentiated iPSCs) in engineered spinal cord tissue. For ECM analyses, 17 ECM-associated genes were chosen from the above 500, and functional enrichment was performed. Functions that were chosen for ECM analyses, were associated with at least two out of the 17 ECM genes.

Z- scores for each sample separately were calculated according to RPKM values. Complete RNA data is available under accession number GSE97341.

Calcium imaging

For calcium imaging, the implants were incubated with 10 µM fluo-4 AM (Invitrogen) and 0.1% Pluronic F-127 (Sigma-Aldrich) for 45 min at 37 °C. Implants were then washed in Hank's buffer salts solution (HBSS) and imaged using an inverted fluorescent microscope (Nikon Eclipse TI). Videos were acquired with an ORCA-Flash 4.0 digital complementary metal-oxide semiconductor (CMOS) camera (Hamamatsu) at 2 frames/s. HBSS was used as the external solution. Baseline was recorded for 30 sec, after which depolarization was induced by KCl (50 mM; final concentration 25 mM) or glutamate (200 µM; final concentration 100 µM) solutions.

Injection of solutions was performed *in situ*, allowing a non-interrupted recording of the cellular Ca^{2+} responses. The data was analyzed using ImageJ (NIH) and normalized by dividing each data set by the first value (F/F_0).

Spinal cord hemisection

Hemisection was performed as previously described (*5*). Briefly, mice (20-30 g) were anaesthetized with intraperitoneally-injected ketamine (100 mg/kg) and xylazine (16 mg/kg) in PBS. The spinal cord was exposed at the low thoracic to high lumbar area. After laminectomy, a complete left hemisection was made at T10 and the overlying muscle and skin were sutured. In acute phase injury, mice were immediately treated. The controls groups were: Untreated group which was treated with 10 uL saline; Cells group, treated with dissociated iPSCs-derived spinal cord neurons (day 30 of differentiation) suspended in 10 µL saline; and Hydrogel group that was treated with 0.75% pre-crosslinked omentum-based hydrogel. The tested treatment was applied to the Implants group which was treated with differentiated iPSCs-derived spinal cord neuron implants (day 30 of differentiation).

In chronic phase injury model, SCI was induced similarly to the acute phase. Six weeks after the initial SCI, animals were re-anesthetized, and the lesion area was re-opened. Scar was identified according to tissue color and texture and carefully resected. Treatments were applied in the cavity formed by the ablated scar. Mice were randomly assigned to the four groups and allowed to survive for 1 week to 3 months post injury.

All mice were treated according to ethical regulations of Tel Aviv University. Permission was granted by the ethical committee, protocol number 04-19-047- “treatment of biological

hydrogel and human neural cells on neuronal regeneration after acute and chronic spinal cord injury in mouse model".

Tissue preparation and immunofluorescence labeling. One-, eight- or twelve-weeks post-treatment, the animals were anesthetized and transcardially perfused with 20 mL PBS (pH 7.4) followed by 20 mL 4% paraformaldehyde. Spinal cord tissue was dissected, post-fixed in 4% paraformaldehyde for 24 h at 4 °C and dehydrated with 20% v/v sucrose overnight at 4 °C. The dissected tissues were embedded in OCT and cut longitudinally into 60-μm thick cryosections using a freezing microtome (Leica CM1950, Germany).

For immunostaining, sections were permeabilized with 0.3% Triton X-100 in PBS solution, blocked with 10% FBS and 1% BSA in PBS for 1 h, and then incubated with primary antibodies (Antibody list, Supporting Information) overnight at 4 °C. After rinsing with PBS three times, the sections were incubated with Alexa Fluor 488/594/647 conjugated secondary antibodies (Antibody list, Supporting Information) for 1 h. Then, the sections were washed for three times with PBS, counterstained with Hoechst 33528 (5 μg/mL) for 10 min at room temperature, then washed with PBS, let dry and covered with glass slips in an anti-fade fluorescent mounting medium. The sections were stored at 4 °C until characterized using a Nikon confocal microscope.

The integrated density of each epicenter was automatically calculated by ImageJ. Pixel/(100 μm)^2 = integrated density/unit area. At least three sections near the epicentral part (at the injury site, from dorsal to ventral) of the spinal cord were investigated for each animal.

Anterograde axonal tracing. Axonal regeneration was examined using anterograde tracing (untreated N=7, cells N=7, hydrogel N=10 and implants N=10). Three months after SCI,

Tetramethylrhodamine dextran (TMRD ;“Fluoro-Ruby”, MW 10,000kD; Molecular Probes) was injected into the spinal cord at the level of the cervical enlargement, ipsilateral to the lesion (*5*).(*5*) After 14 days, mice were perfused with PBS followed by 4% paraformaldehyde (PFA). Spinal cords were removed and post-fixed for 1 hour in cold 4% PFA followed by 20% sucrose in PBS overnight at 4 °C. Longitudinal (horizontal) serial cryostat sections were cut (60 µm) and slides were imaged using fluorescence microscopy (Nikon ECLIPSE NI-E). Labelled axons in the white matter were quantified at 1000 µm, 500 µm, 200 µm rostral to the lesion site and 100 µm caudal to the lesion site at 400x. Photomontage of the regenerating axons was taken using fluorescence microscopy.

Catwalk gait analysis

Gait measurements were collected using the CatWalk XT system (Noldus Information Technology, The Netherlands). Data were transmitted to a computer and analyzed with the CatWalk XT® software (version 10.6, Noldus). Each mouse was located on one side of the walkway and had to complete 3 compliant runs (variation < 60%; time < 5 sec) to the other side. The coordination (regularity index) and the ability of the mouse to put pressure on the injured paw (left hind max intensity mean) were tested. The parameters were calculated for every run and the results were averaged for every time point per animal.

Grid walk

The mice were tested walking through a horizontal grid (1.2 × 1.2–cm grid spaces, 35 × 45–cm total area), one week prior to the treatment and on weeks 1, 2, 4, 6, 8 post treatment. Each mouse was allowed to walk freely on the grid for 3 minutes. When the left hindlimb paw protruded entirely through the grid with all toes and heel, it was counted as a misstep. The

number of missteps and total number of steps taken with the left hindlimb were both counted. The results were expressed as a percentage of correct steps on left hind paw (*6*).

Magnetic resonance imaging (MRI)

MRI was performed on weeks 1 and 4 post chronic scar resection by a Bruker Biospec 7T/30 Scanner equipped with a 660mT/m gradient unit, using a cross coil configuration of 86mm transmissive volume coil and 10mm loop coil as a receiver. For untreated and cells groups N=4, for hydrogel and implants groups N=5. Animals were under 1-2% isoflurane in O_2 anesthesia on a heating pad, with breathing monitored and body temperature maintained at 37 °C.

MRI protocol included the following methods: T2 weighted (T2w) images that were acquired using the rapid acquisition with relaxation enhancement (RARE) sequence and Diffusion Tensor Imaging (DTI) acquisition with a Diffusion-Weighted Spin-Echo Echo-Planar-Imaging pulse sequence (DW-SE-EPI). T2w acquisition was performed with the following parameters: TR=8000 ms; effective TE: 30 ms, RARE factor 12 with 3 repetitions. 32 axial slices, 0.45 mm thick (no gaps), with in-plane resolution of 0.15 mm^2 covering the entire cerebrum, for 4 min. For DTI, performed for 5.5 min, the following parameters were used: TR/TE=2500/19.2 ms, Δ/δ=10/2.5 ms, 2 EPI segment, 30 gradient directions with b-value at 1000 s/mm2 and three B0 images, 30 axial slices, 0.45 mm thick (no gaps), in-plane resolution was 0.30 mm^2. The total MRI protocol acquisition took ~20 min. ExploreDTI software was used for DTI calculations and fiber tracking. The eigen-components decomposed from the tensors were used for calculating fractional anisotropy maps. Regions of interest of the spinal cord were manually segmented in each slice. Fiber tracking was employed for tract orientation with angle <30° and FA <0.15 and resolution of 2 2 2.

Statistical analysis

All statistical analyses were performed using GraphPad Prism 8.00 (GraphPad Software, Inc., USA). Data are shown as mean ± SEM (Standard Error of the Mean). Data were analyzed using Student's t-test or one-way analysis of variance (ANOVA) followed by Tukey's post-hoc test. The values were considered significantly different at $p < 0.05$.

Rheological properties

Rheological measurements (n=3; individual samples) were taken using Discovery HR-3 hybrid Rheometer (TA Instruments, DE) with 8 mm diameter parallel plate geometry with a peltier plate to maintain the sample temperature. The samples were loaded at a temperature of 4 °C, which was then raised to 37 °C to induce gelation; during which the oscillatory moduli of samples were monitored at a fixed frequency of 1 Hz and a strain of 1%. After gelation the same samples were monitored using a frequency sweep (0.1-10 Hz) under 1% strain. Cellular constructs were crosslinked prior to the rheology and were tested at different frequencies as mentioned above.

Scanning electron microscopy

To prepare the samples (either cellular or acellular), the constructs were fixed with 2.5% glutaraldehyde (16-20 hr at 4 °C), followed by graded series of ethanol–water solutions for dehydration (25–100%). All samples were critical point dried, mounted onto aluminum stubs with conductive paint and sputter-coated with gold in a Polaron E 5100 coating apparatus (Quorum technologies, Lewis, UK). Samples were observed under JCM-6000PLUS NeoScope Benchtop (JEOL USA Inc., Peabody, MA).

Flow cytometry

For flow cytometry analysis, cells were isolated from implants using up to six cycles (30 min each) of enzyme digestion with collagenase type II (95 U/mL; Worthington, Lakewood, NJ) and pancreatin (0.6 mg/mL; Sigma–Aldrich) in Dulbecco's modified Eagle Medium (DMEM, $CaCl_2 \cdot 2H_20$ (1.8 mM), KCl (5.36 mM), $MgSO_4 \cdot 7H_2O$ (0.81 mM), NaCl (0.1 M), $NaHCO_3$ (0.44 mM), NaH_2PO_4 (0.9 mM)). After each round of digestion cells were centrifuged (120 g, 5 min) and re-suspended in DMEM/D12 and kept on ice. Cells were washed in PBS and then treated with Accutase (Stemcell Technologies, Vancouver. Canada) for 5 min at 37 °C following mechanical trituration to assure dissociation to single cells.

For membrane proteins, cells were stained with conjugated antibody or isotype control for 30 min at RT.

For intracellular proteins, cells were fixed with 4% formaldehyde, washed with PBS, permeabilized with triton 0.1% and incubated with primary and thereafter secondary antibodies for 30 min each, on ice. Cells were analyzed and data analysis was performed using CytoFlex 4 flow cytometer (Beckman Coulter, USA). Positive populations were gated according to unstained cells and appropriate isotype control. At least 3 biological replicates were analyzed.

Antibody list

Primary antibodies: OCT4 (ab27985, 1:100), Ki67 (ab16667, 1:250), TUJ1 (ab7751/ab18207, 1:500), MAP2 (ab5392, 1:1000), NFM (ab24574, 1:1000), SYP (ab32127, 1:500), Iba1 (ab178846, 1:400); Nestin (ab134017; 1:2000); netrin1 (ab37390; 1:100), slit1 (ab115892; 1:100) and Cytopainter red (ab138893) have been acquired from Abcam (Cambridge, MA). GFAP (DAKO Z0334, 1:1000); NeuN (MAB377; 1:200; Millipore); Collagen I (MA1-26771, 1:2000), and TMRD

(dextran, tetramethylrhodamine, D1817, 10000MW) have been acquired from Invitrogen. HB9 (81.5C10, 1:100) was purchased from DSHB.

Secondary antibodies: Alexa Fluor 488 (1:250; 111-545-003; Jackson); Alexa Fluor 555 (1:500; ab150118, Abcam), Alexa Fluor 647 (1:500; ab150135/ab150175, Abcam) . Nuclei were visualized with Hoechst33258 (5 µg/mL).

Conjugated antibodies: TRA-1-60-PE (1:100; 130-122-921, Miltenyi, Germany); SSEA-4 (1:100; 130-122-918, Miltenyi);

Acute: untreated and cells groups n=7/group, hydrogel and implants groups n=10/group. Chronic: untreated and cells groups n=5/group, hydrogel and implants groups n=6/group.

1. M. Shevach *et al.*, Omentum ECM-based hydrogel as a platform for cardiac cell delivery. *Biomedical materials* **10**, 034106 (2015).
2. R. Edri *et al.*, Personalized hydrogels for engineering diverse fully autologous tissue implants. *Advanced materials* **31**, 1803895 (2019).
3. M. Martin, Cutadapt removes adapter sequences from high-throughput sequencing reads. *EMBnet. journal* **17**, 10-12 (2011).
4. S. Anders, P. T. Pyl, W. Huber, HTSeq—a Python framework to work with high-throughput sequencing data. *bioinformatics* **31**, 166-169 (2015).
5. Y. Goldshmit, M. P. Galea, G. Wise, P. F. Bartlett, A. Turnley, Axonal regeneration and lack of astrocytic gliosis in EphA4-deficient mice. *J Neurosci* **24**, 10064-10073 (2004).
6. Y. Goldshmit, E. Banyas, N. Bens, A. Yakovchuk, A. Ruban, Blood glutamate scavengers and exercises as an effective neuroprotective treatment in mice with spinal cord injury. *Journal of Neurosurgery: Spine SPI* **33**, 692-704 (2020).

Supplementary Figures

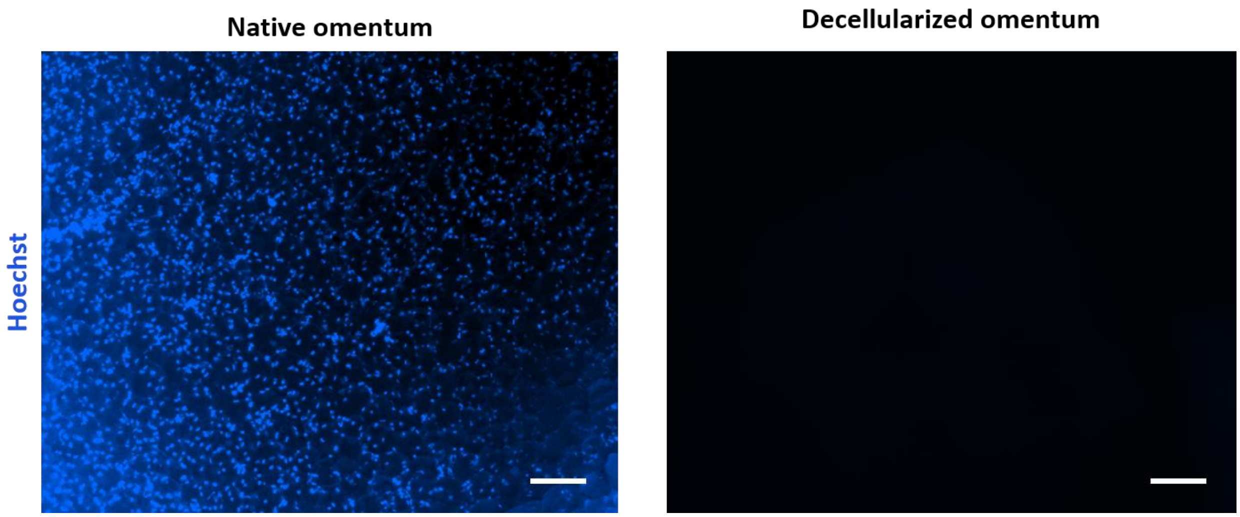

Figure S1. Nuclei staining of native (left) and decellularized (right) omentum under the same exposure conditions. Scale bar=200 µm.

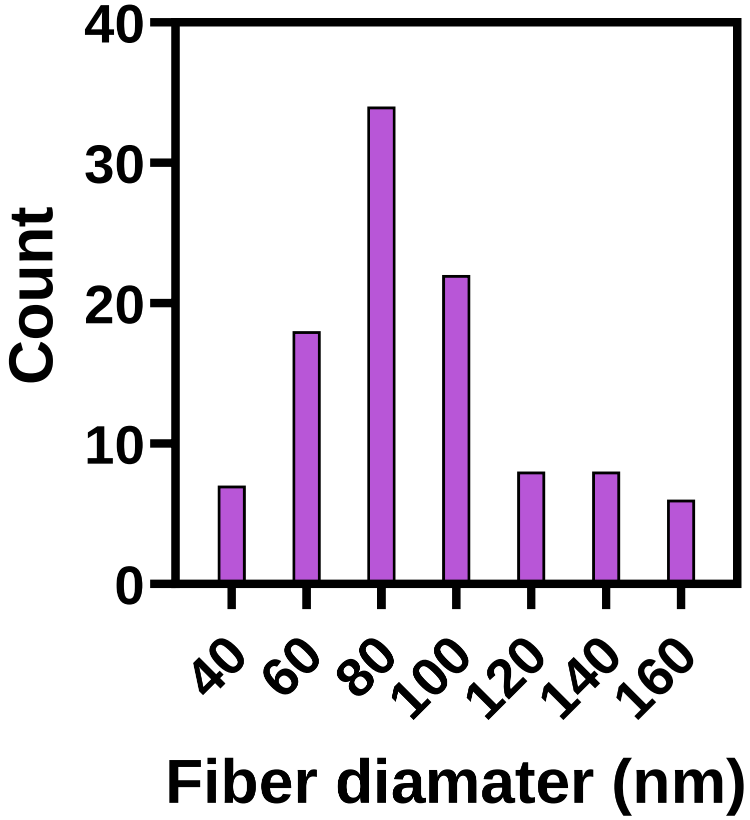

Figure S2. Fiber diameter histogram of omentum-based hydrogel.

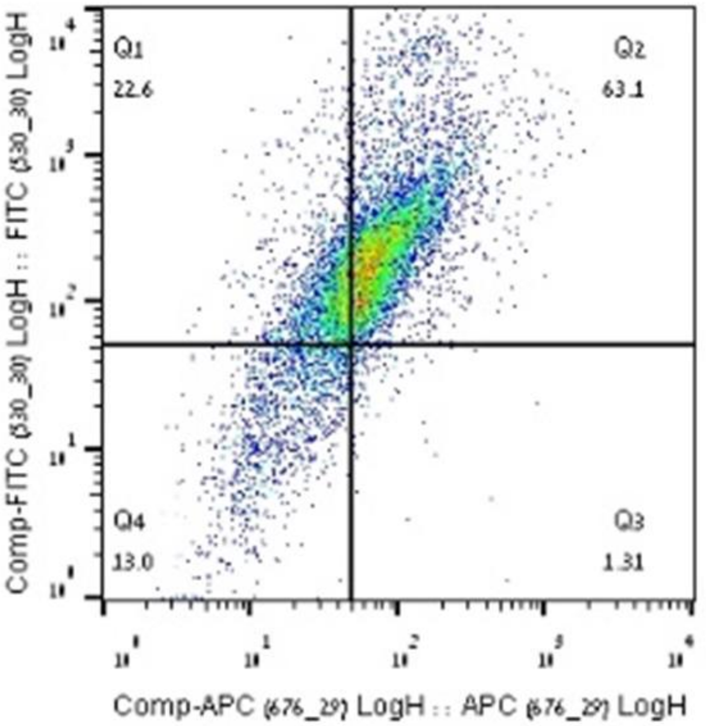

Figure S3. Representative flow cytometry analysis of iPSCs-derived spinal cord neurons. The cells were double-stained for neural marker beta-III tubulin (TUJ1) and specific spinal cord marker (HB9).

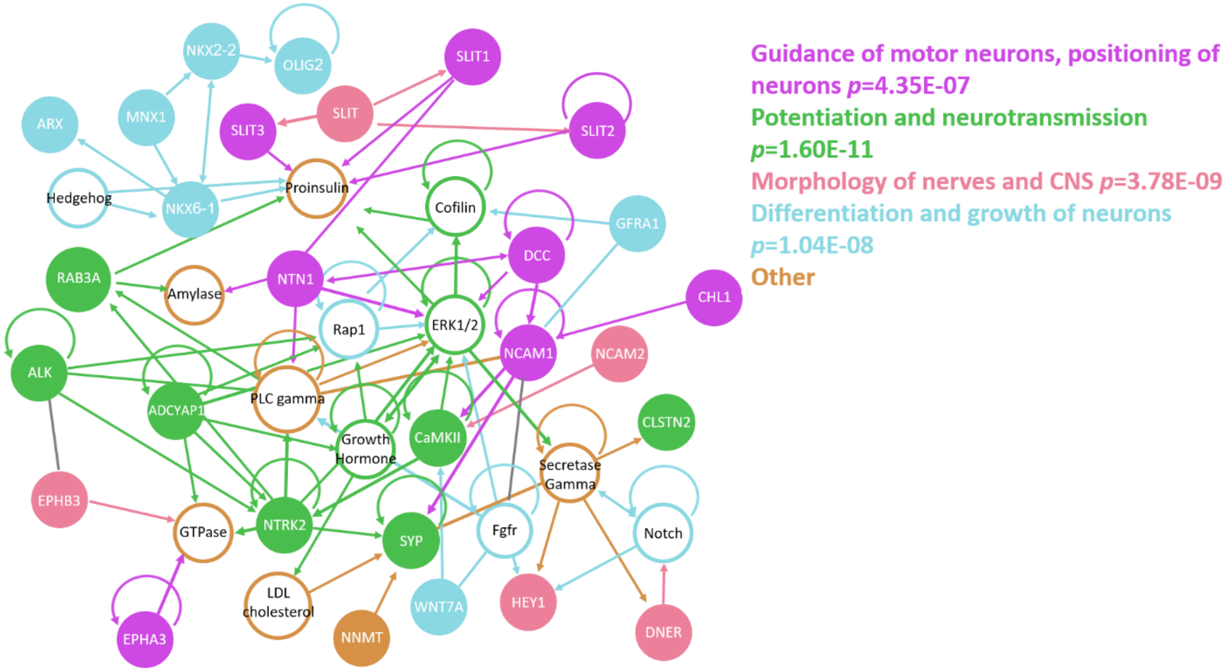

Figure S4. Network analysis at day 30 of differentiation based on RNA seq using the ingenuity pathway analysis tool. The network analysis was applied on the 500 top increasing genes during the differentiation process. Functions of neuronal activity and maturation are significantly enriched in the resulting network (color coded). *p* indicates enrichment *p*-values.

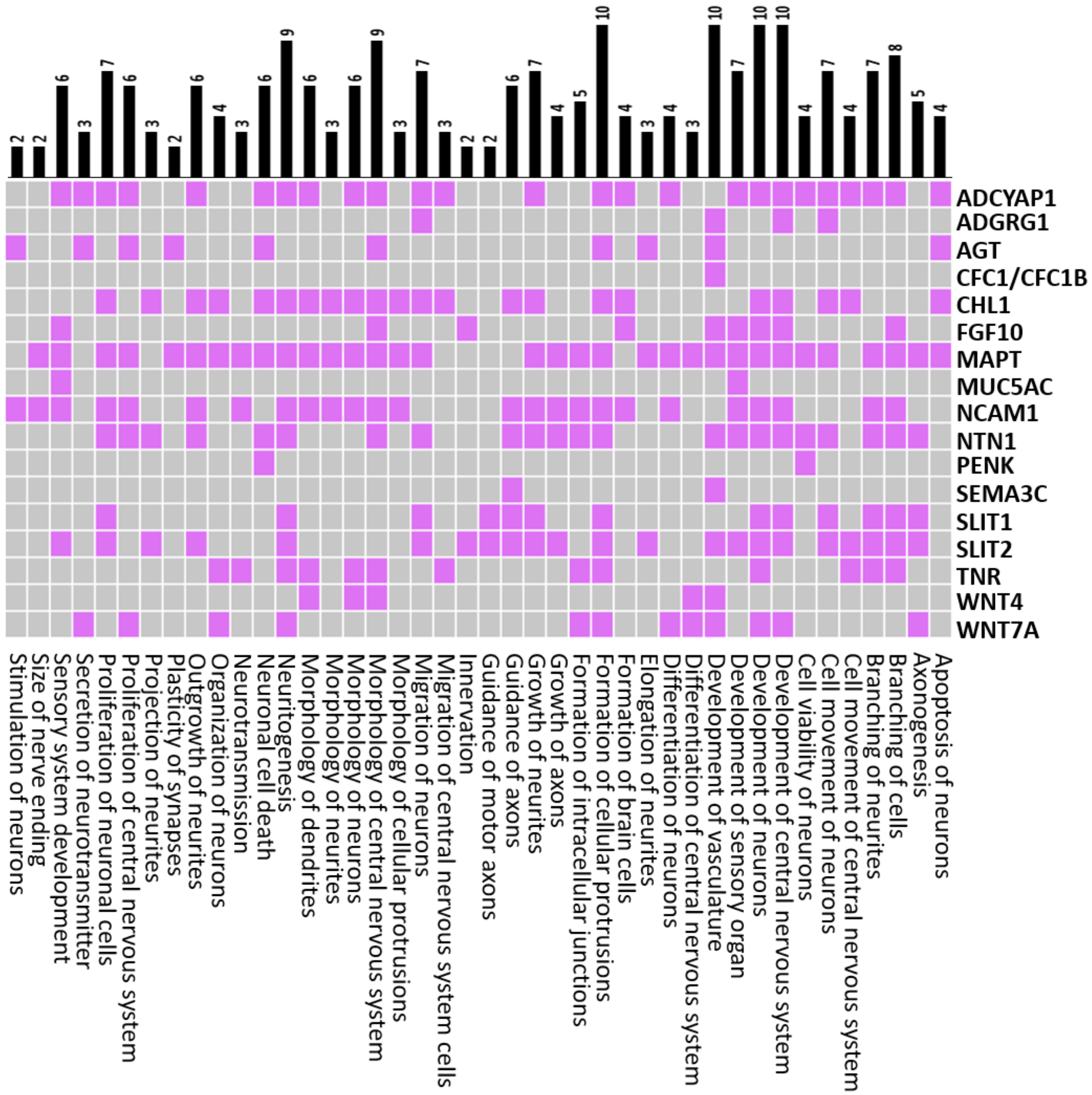

Figure S5. Table of 17 ECM genes and associated enriched functions. Purple indicates that the gene is associated with the enriched function. The bars indicate the number of ECM genes associated with a specific function.

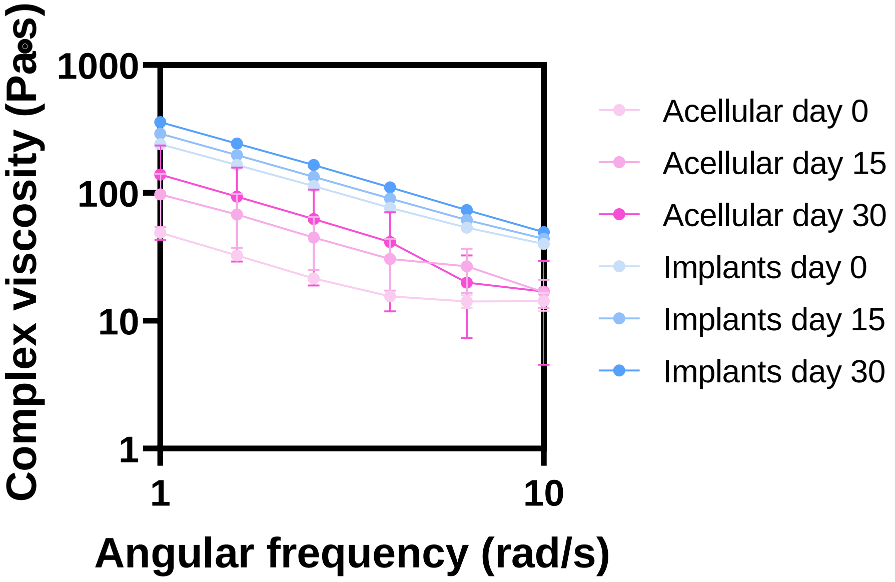

Figure S6. Complex viscosity at different frequencies of acellular hydrogel and implants at days 0, 20 and 30 of differentiation.

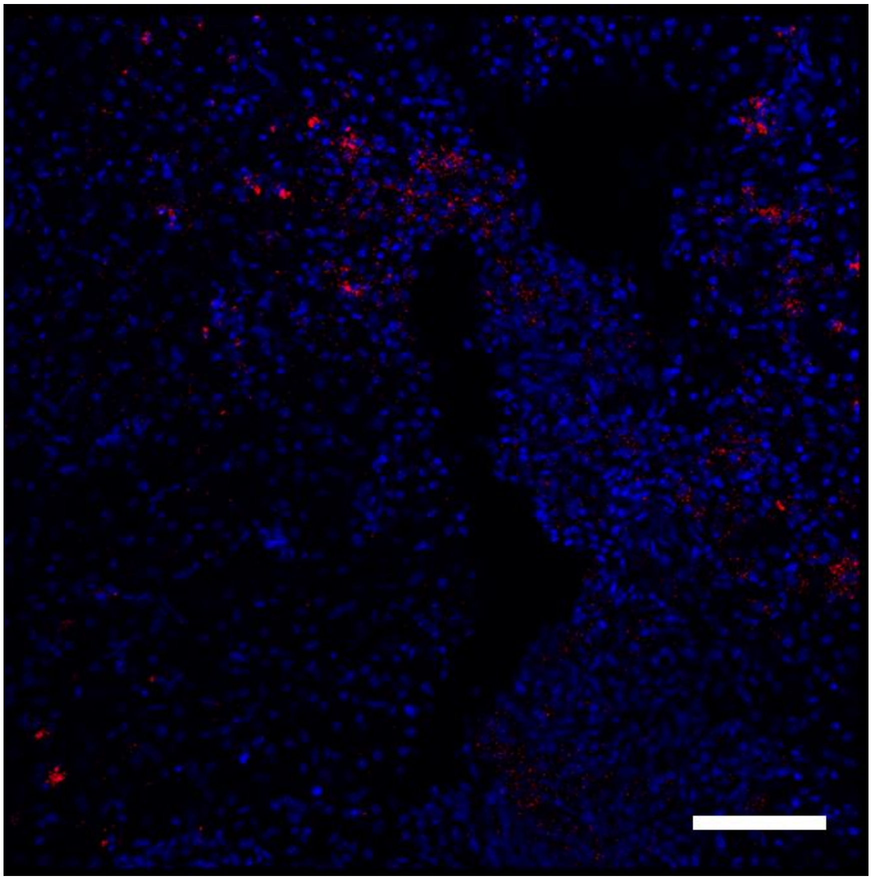

Figure S7. Cell engraftment of the injected cells group, 7 days post treatment. Cells were labeled with cytopainter (red) prior to transplantation. Nuclei appear in blue (Hoechst). Scale bar=100 µm.

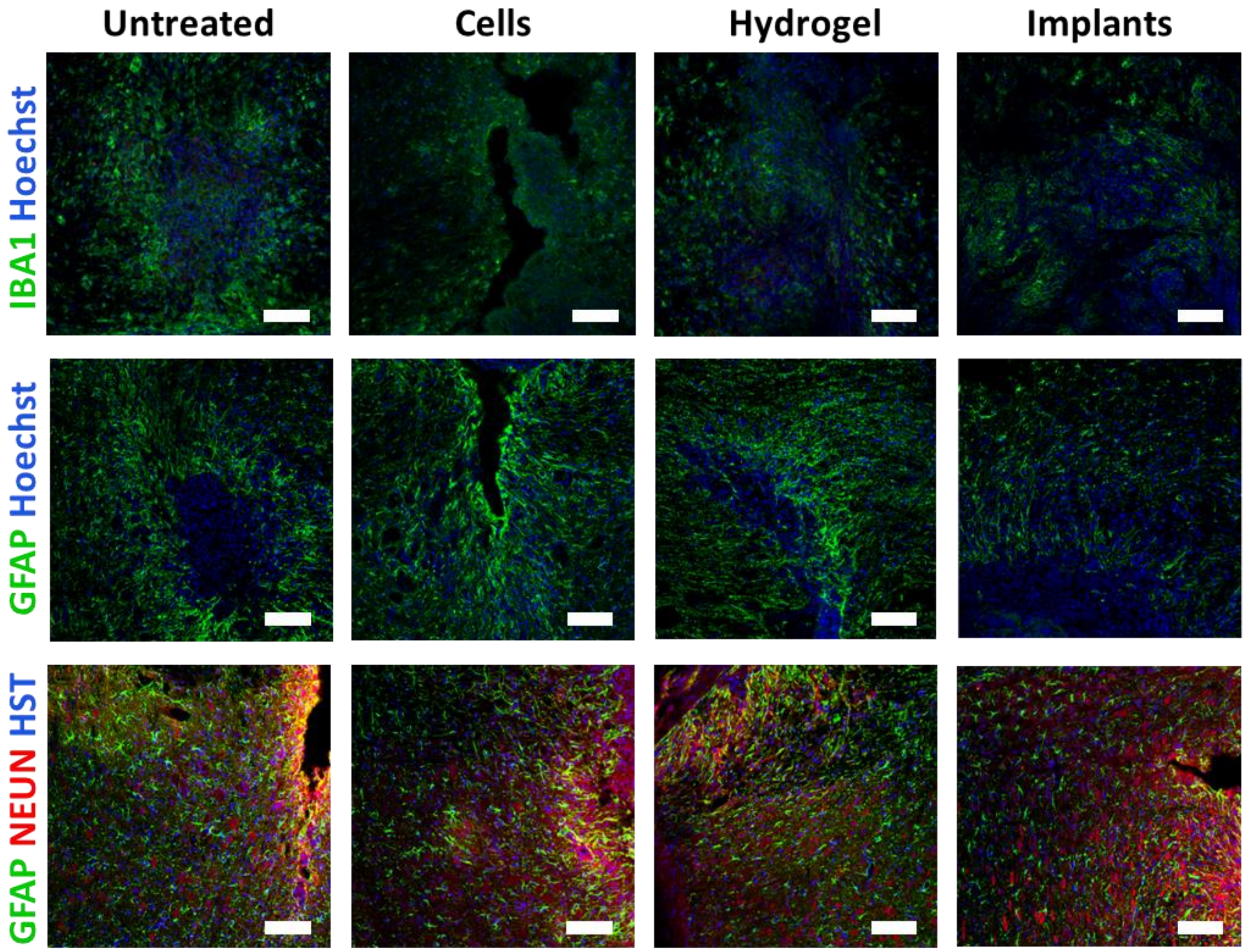

Figure S8. Representative images of immunolabeling at lesion site in acute model, 7 days post treatment. Scale bar=100 µm. Upper panel- Microglia (IBA1); Middle panel- Astrocyte (GFAP); Lower panel- Neuronal nuclei (NeuN in red) and astrocytes (GFAP in green).

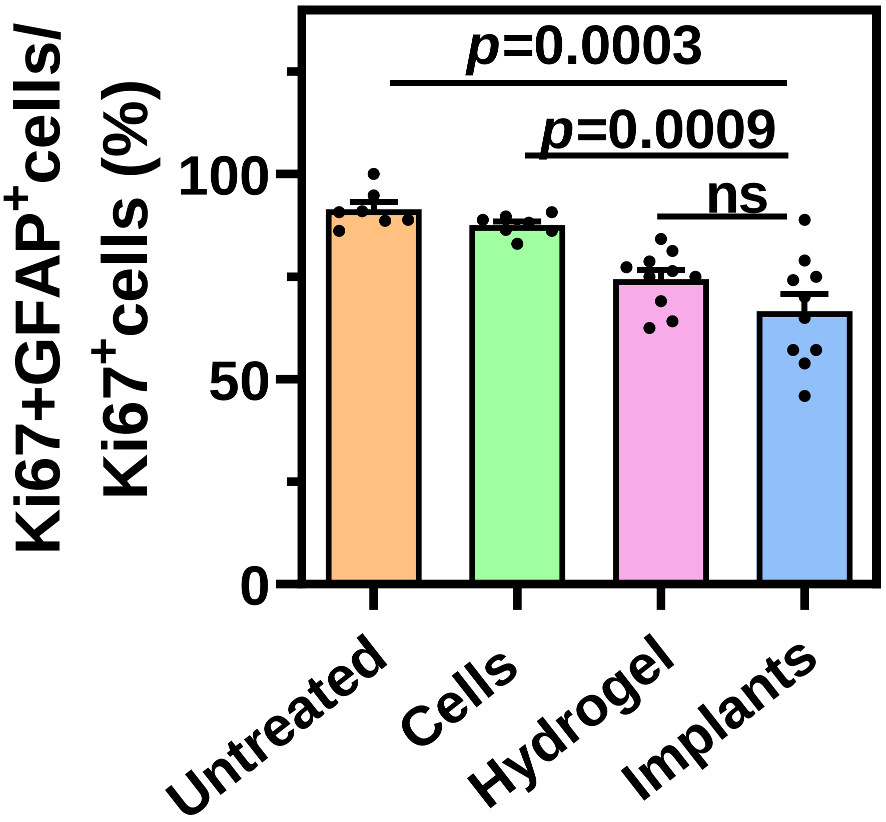

Figure S9. Proliferative astrocytes. Double positive staining of Ki67 proliferative marker and GFAP (astrocytes).

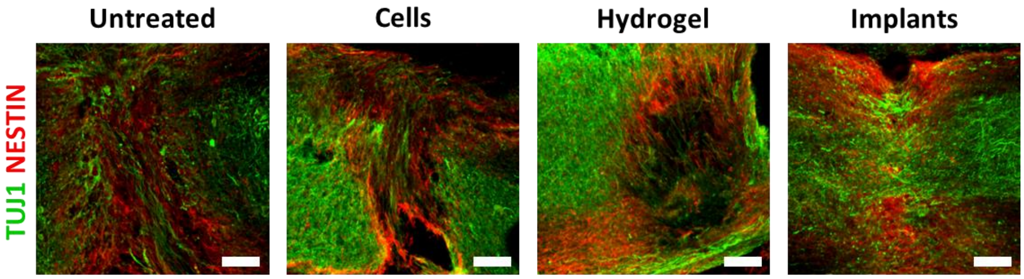

Figure S10. Immunostaining of neural stem cells (NESTIN) and neurons (TUJ1) at the lesion site of animals treated at the acute phase 12 weeks post implants treatment. Scale bar=100 µm.

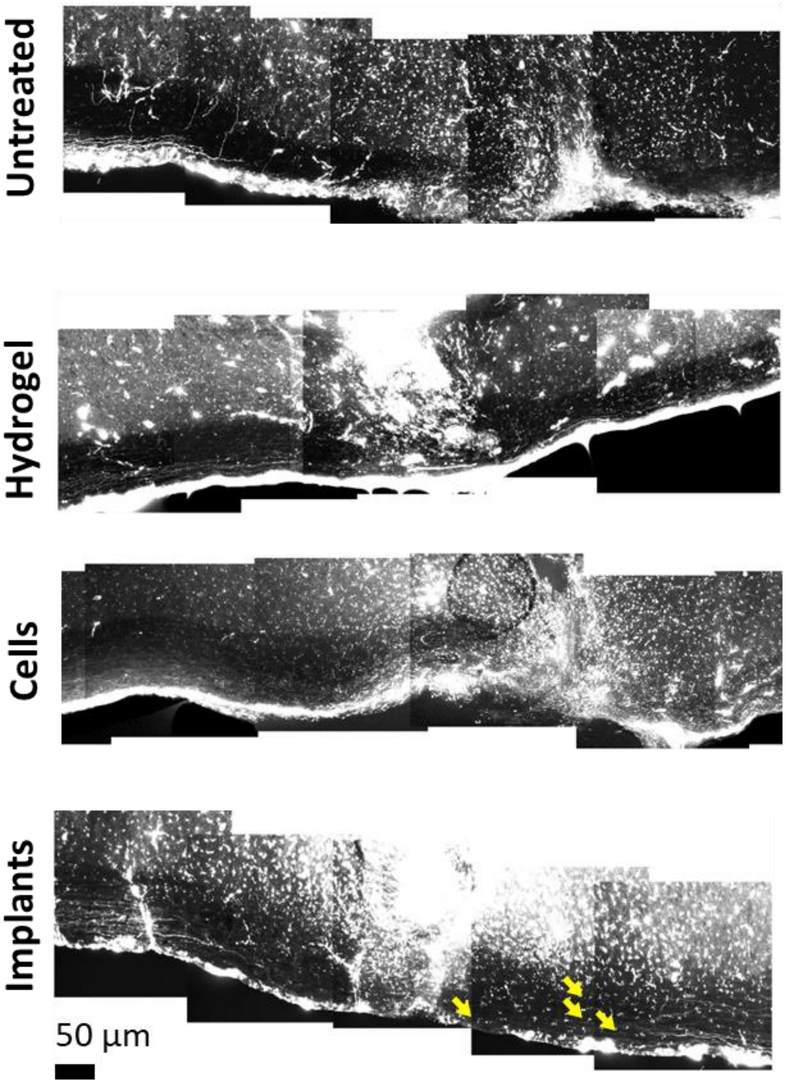

Figure S11. Anterograde tracing. Photomontage of anterograde tracing by TMRD. Yellow arrows indicate on axons at or caudally to the lesion site. Scale bar= 50 µm.

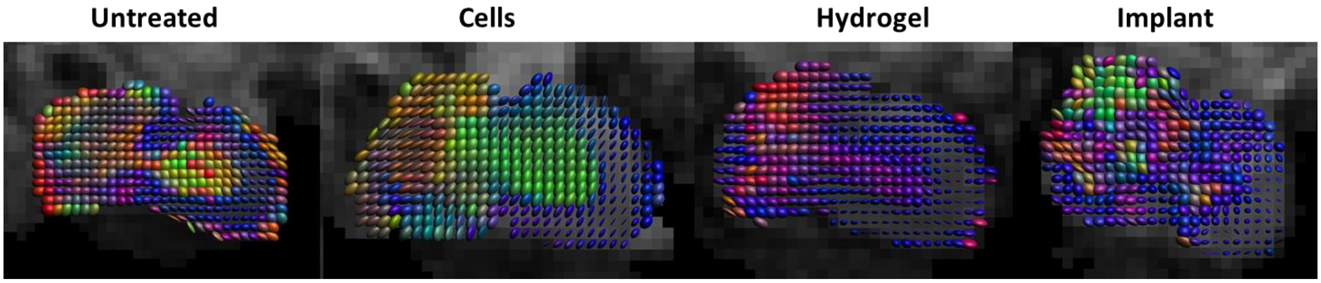

Figure S12. Glyph-based visualization of diffusion tensor imaging at 1 week after scar resection and post treatment. Glyphs are presented on the background of axial diffusion tensor images. Blue indicates fibers in the rostral-caudal axis, red indicates right and left orientation both medial and lateral, while green indicates anterior to posterior orientation.

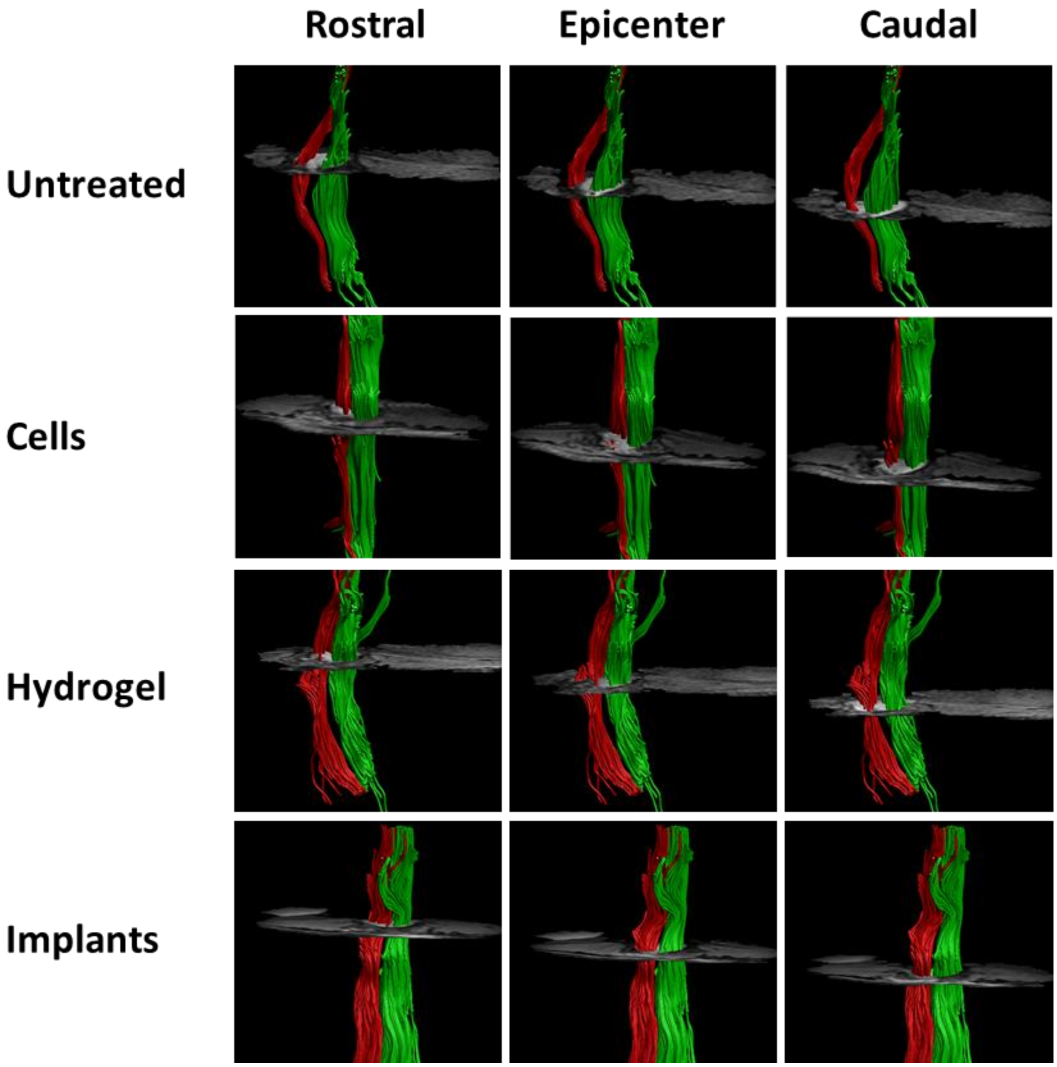

Figure S13. Fiber tractography 4 weeks post scar resection and treatment. Fiber tractography reconstructed in the axial plane. Red fibers are ipsilateral to the initial hemisection, and green fibers are contralateral (unharmed). The fibers are shown from frontal view, with axial slices at the epicenter, rostral (+0.6 mm above lesion) and caudal (-0.6 mm below lesion).

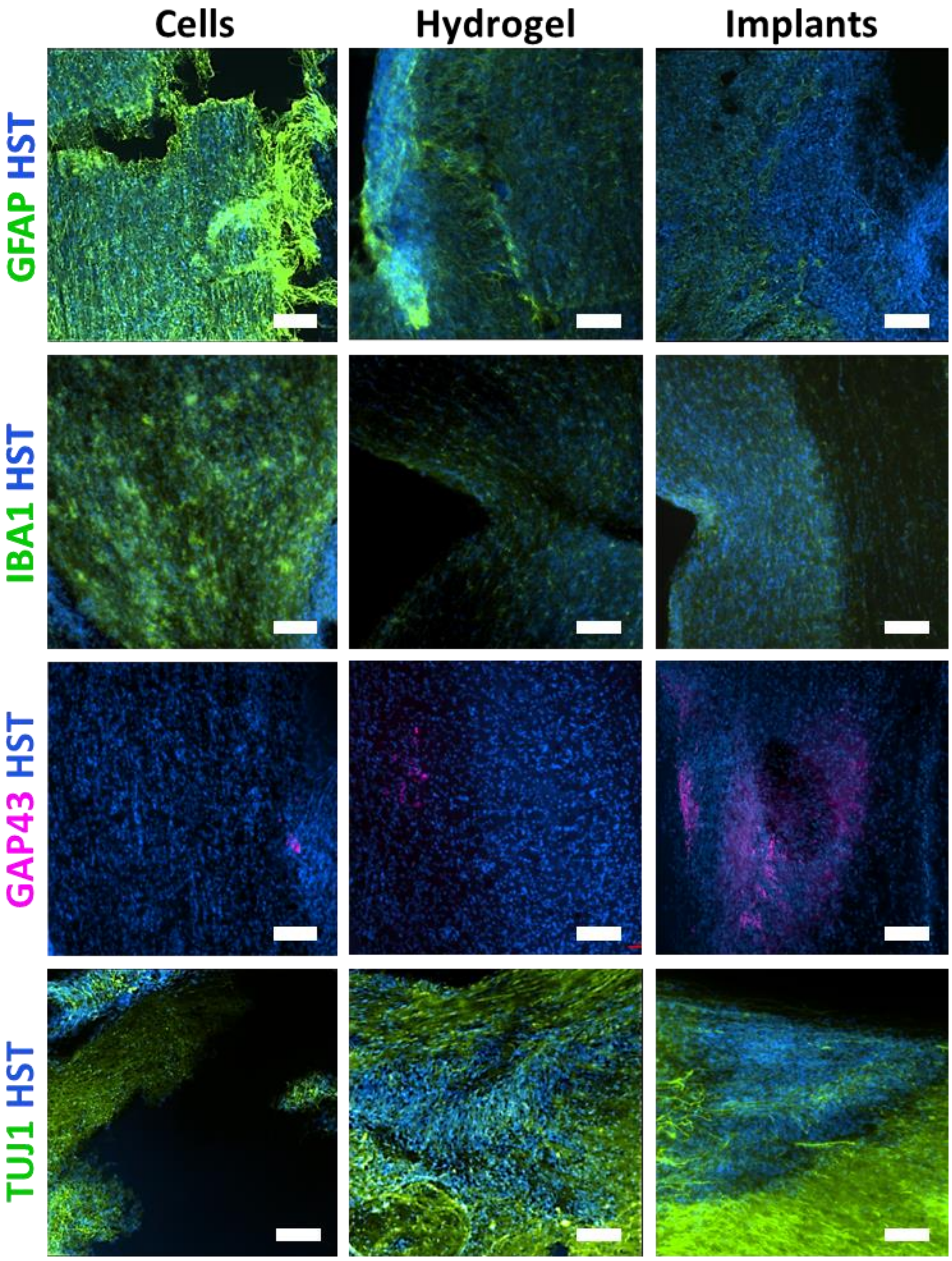

Figure S14. Cellular content 8 weeks post treatment at the chronic phase. Immunostaining of astrocytes (GFAP), microglia (IBA1), nerve growth associated protein (GAP43) and neurons (TUJ1) at the lesion site. Scale bars= 100 µm.

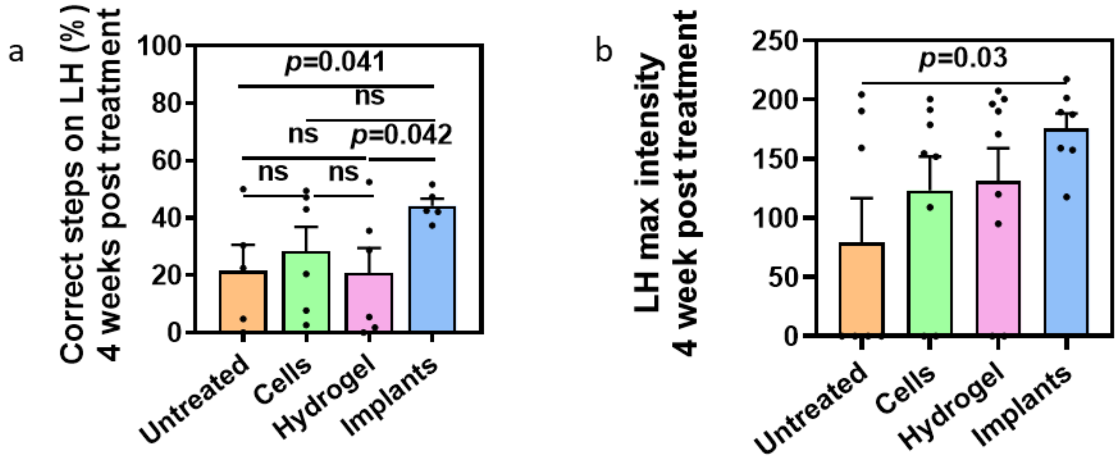

Figure S15. Behavioral studies 4 weeks post chronic scar resection and treatment. a. Grid walk testing the sensorimotor of the mice. b. Left hind max intensity, correlating to the pressure the mice place on the injured foot.
